# A multifunctional ZIF-8@CuO/collagen-binding peptide hydrogel for photothermal-enhanced antibacterial and infected skin wound management

**DOI:** 10.1039/d6ra06950a

**Published:** 2026-10-08

**Authors:** Shengyang Li, Renfeng Zhang, Haorui Du, Hanwen Yang, Su Pan

**Affiliations:** a Department of Orthopedics, The Second Hospital of Jilin University Yatai Street 4026, Nanguan District Changchun 130022 Jilin Province China pansu@jlu.edu.cn

## Abstract

Owing to continuous bacterial proliferation, a persistent inflammatory response, and impaired vascularization, infected skin wounds remain a major clinical challenge. Herein, we fabricated a multifunctional skin-repair material by incorporating ZIF-8@CuO nanoparticles and a collagen-binding vascular endothelial growth factor mimetic peptide (C-VEGFMP) into a gelatin methacryloyl (GelMA) hydrogel. The ZIF-8@CuO nanoparticles were prepared by controllably depositing copper oxide (CuO) onto the surface of zeolitic imidazolate framework-8 (ZIF-8). ZIF-8@CuO can endowed the hydrogel with potent antibacterial and anti-inflammatory activities, as well as efficient near-infrared (NIR) photothermal conversion capability. C-VEGFMP was constructed by introducing a collagen-binding sequence into the VEGFMP. This modified peptide can stably anchor to GelMA and maintain its bioactivity at the hydrogel–tissue interface. The resulting ZIF-8@CuO/C-VEGFMP hydrogel exhibited favorable cytocompatibility, broad-spectrum antibacterial activity and prominent pro-angiogenic performance. Upon NIR irradiation, the photothermal effect of ZIF-8@CuO can synergistically eradicate pathogenic bacteria, thereby enhancing photothermal therapeutic efficacy. In addition, the sustained presence of bioactive C-VEGFMP promoted epithelial regeneration, collagen deposition, and neovascularization in infected full-thickness wounds. Transcriptomic analysis indicated that treatment with ZIF-8@CuO/C-VEGFMP under NIR irradiation exerted anti-inflammatory, pro-angiogenic and tissue-repair effects mainly by upregulating thrombomodulin (THBD) and Ets proto-oncogene 2 (Ets2) genes. This study provides a multifunctional strategy that combines nanoparticle-mediated photothermal antibacterial effects with functionalized peptides, offering a promising therapeutic modality for the management of infected skin injuries.

## Abstract 2

This schematic illustrates the construction of ZIF-8@CuO/C-VEGFMP GelMA hydrogel and its NIR-activated multifunctional actions for infected full-thickness skin wound healing.

## Graphical abstract

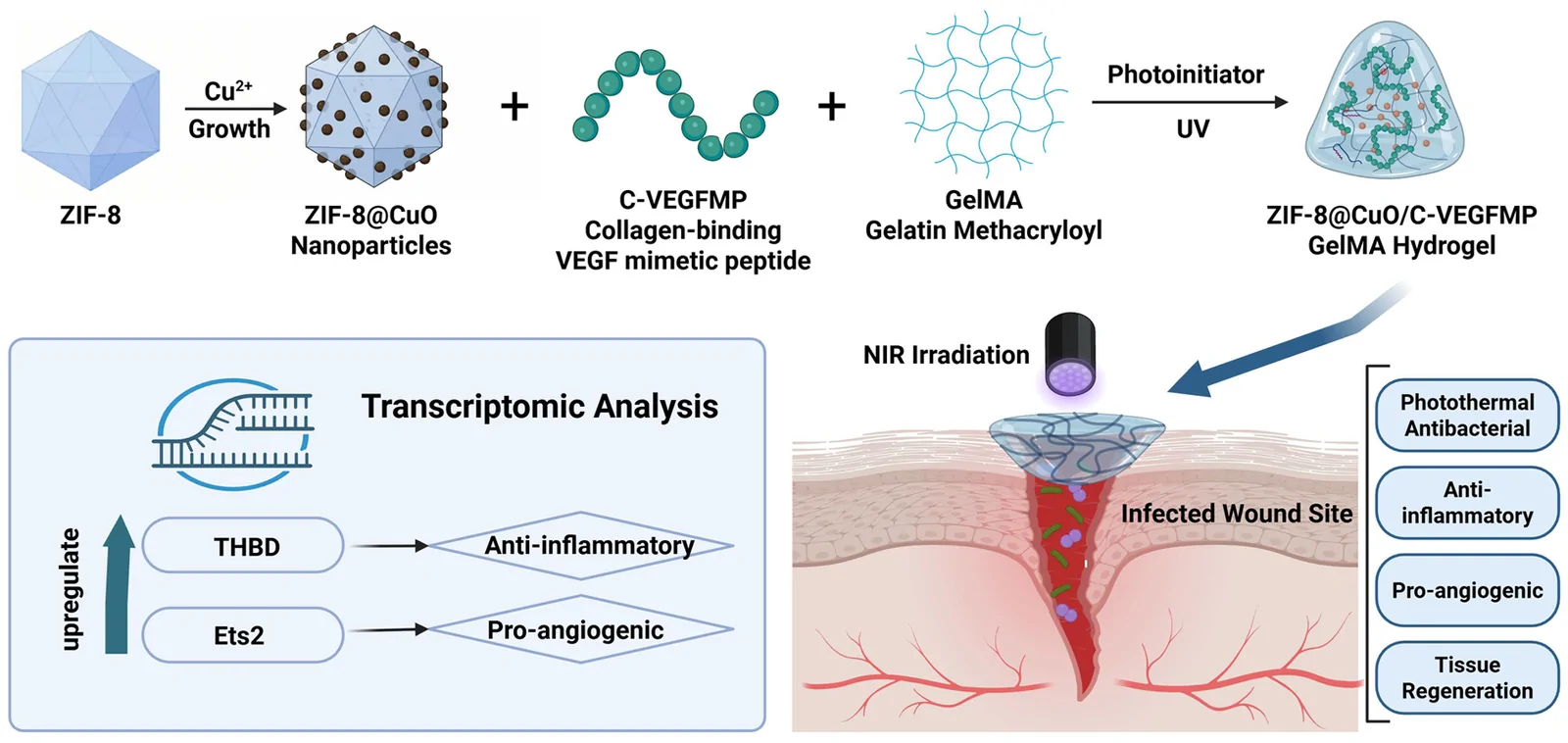

## 1 Introduction

When skin sustains damage from trauma, surgery, burns and other insults, the wound site becomes susceptible to bacterial invasion, which may further trigger local or systemic clinical symptoms.^1,2^ Bacterial infection induces excessive inflammatory responses, elevates reactive oxygen species (ROS) levels, and damages the extracellular matrix, thereby hindering orderly wound healing. The primary objectives for managing infectious skin wounds are anti-infective intervention and adequate physical wound coverage.^3,4^ Nevertheless, overreliance on antibiotics has promoted the emergence of drug-resistant bacteria such as methicillin-resistant *Staphylococcus aureus* (MRSA), thereby increasing treatment complexity and complicating infection control.^5^ Photothermal therapy has been explored for treating infectious skin wounds.^6,7^ This approach generates local overheating through physical mechanisms, thereby eradicating pathogens and circumventing conventional antibiotic-dependent pathways, while reducing the risk of drug-resistant bacteria. However, effective photothermal therapy often requires prolonged exposure to higher temperatures, which may cause thermal damage to normal tissues around the wound. Therefore, photothermal agents are required to precisely target the damaged area to reduce adverse reactions. Combining photothermal effects with other bactericidal pathways represents a promising strategy to lower the required therapeutic temperature and mitigate thermal side effects. Among diverse nanofillers, MOF-derived and coordination-polymer nanoparticles are particularly attractive owing to their adjustable metal-ion release and multimodal bactericidal capability. Various MOF-derived and coordination-polymer nanoparticles have been incorporated into hydrogel dressings for infected wound therapy, benefiting from their metal-ion release and multi-modal antibacterial activity, which has been comprehensively reviewed elsewhere.^8^ To overcome the limitations of conventional therapeutic strategies for infectious skin injuries, there is an urgent need to develop multifunctional skin-repair biomaterials.

Among various candidate materials, ZIF-8 and CuO have attracted particular attention because of their complementary antibacterial and photothermal properties. Zeolitic imidazolate framework 8 (ZIF-8) has demonstrated great potential in biomedical applications due to its high porosity, large specific surface area, and capacity to modulate Zn^2+^ release upon degradation, which confers antibacterial and tissue-regenerative effects.^9,10^ However, ZIF-8 alone often lacks sufficient antibacterial potency to eradicate established infections, and it possesses no light-to-heat conversion capability. In comparison, copper oxide (CuO) is a low-cost metal oxide with broad-spectrum antibacterial activity, intrinsic enzyme-mimicking properties, favorable inflammatory-microenvironment-regulating capacity, and strong near-infrared (NIR) absorbance that enables efficient photothermal conversion.^11–13^ CuO also exhibits antioxidant activity, effectively eliminating 2,2-diphenyl-1-picrylhydrazyl (DPPH) free radicals by catalyzing the degradation of hydrogen peroxide.^14,15^ Importantly, CuO can promote angiogenesis and tissue regeneration.^16,17^ Accordingly, integrating CuO with ZIF-8 can yield a multifunctional nanomaterial featuring superior stimuli–responsive antibacterial performance (pH- and NIR-responsive), antioxidant activity, as well as pro-angiogenic and tissue-regenerative functions.

In addition to antibacterial and photothermal functions, adequate angiogenesis is essential for skin regeneration. Restoring functional vascular networks is a critical step for skin regeneration, and vascular endothelial growth factor (VEGF) serves as the master regulator of angiogenesis.^18,19^ VEGF-mimetic peptides (VEGFMP) are polypeptides that recapitulate the biological functions of VEGF and can efficiently drive tissue angiogenesis.^20,21^ Furthermore, VEGFMP exhibit superior performance over full-length VEGF regarding functional modification and thermal stability. Accordingly, numerous studies have improved the skin-repair performance of biomaterials by incorporating VEGF-mimetic peptides into their matrix.^22,23^ Nevertheless, the therapeutic utility of VEGFMP is frequently restricted by drawbacks including short half-life, fast diffusion away from wound sites, weak material-binding stability, and potential off-target effects, all of which compromise their therapeutic efficacy. In recent years, with the development of genetic engineering technology, the method of introducing specific protein functional sequences into polypeptides and growth factors to enhance the material binding ability has been widely applied. For example, Wang *et al.* introduced a chitin binding domain (ChtBD) into basic fibroblast growth factor (bFGF). The experimental results showed that incorporating the ChtBD significantly enhanced the binding ability of bFGF and chitin membranes, enabling cells to adhere and proliferate more effectively.^24^ These findings demonstrate that installing specific binding sequences into growth factors or peptides can greatly improve their material-binding stability, avert premature cargo release, and sustain local pro-angiogenic stimulation at the material–tissue interface during skin wound healing.

Thus, we fabricated a multifunctional skin-repair hydrogel by incorporating ZIF-8@CuO nanoparticles and collagen-binding VEGFMP (C-VEGFMP) into gelatin methacryloyl (GelMA) hydrogel. Within this integrated system, ZIF-8@CuO nanoparticles endow the hydrogel with antibacterial, anti-inflammatory, and outstanding photothermal–conversion properties. Meanwhile, C-VEGFMP can stably bind to the collagen motifs in the GelMA network and sustain biological activity at the wound–tissue interface over a prolonged period. Finally, we further validated the therapeutic performance of the ZIF-8@CuO/C-VEGFMP hydrogel combined with photothermal therapy using a rat infectious full-thickness skin wound model. This work aims to construct a multifunctional skin repair biomaterial and offer a highly promising therapeutic strategy for infectious skin injuries.

## 2 Materials and methods

### 2.1 Materials

ZIF-8 and CuO were purchased from XFNano (Jiangsu, China). GelMA was purchased from SunP Biological (Beijing, China). VEGFMP (KLTWQELYQLKYKGI), FITC-labelled VEGFMP (FITC-VEGFMP), C-VEGFMP (TKKTLRTKLTWQELYQLKYKGI) and FITC-labelled C-VEGFMP (FITC-C-VEGFMP) were purchased from GL Biochem Ltd (Shanghai, China).

### 2.2 Synthesis and characterization of ZIF-8@CuO and C-VEGFMP

The synthesis steps of ZIF-8@CuO were as follows. Briefly, 0.2 mmol of CuO was dispersed in 25 mL of methanol, followed by the addition of 1 mmol of Zn (NO_3_)_2_·6H_2_O and 60 mg of polyvinylpyrrolidone (PVP); the mixture was ultrasonicated for 20 min to prepare solution A. Solution B was prepared by dissolving 800 mg of 2-methylimidazole in 25 mL of methanol. Solution B was then added to solution A under stirring, and the reaction was allowed to proceed for 12 h. The product was collected, washed, and dried to obtain the ZIF-8@CuO composite. ZIF-8@CuO was characterized using a transmission electron microscope (TEM) and an energy dispersive spectrometer (XL30 ESEM-FEG, FEI Company). The structural and compositional properties were further characterized by Fourier transform infrared (FTIR) spectroscopy (Bruker, Germany), X-ray diffraction (XRD, D8 ADVANCE, Germany), Raman spectroscopy (DXR, Thermo Fisher Scientific, USA), and thermogravimetric analysis (TGA, Q500, TA Instruments, USA). UV-vis spectroscopy (UV-3600 Plus, Shimadzu, Japan) was used to record the absorption spectra.

To compare the binding affinities of C-VEGFMP and VEGFMP to GelMA, FITC-labeled C-VEGFMP and VEGFMP, each at 1 µM, were separately incorporated into GelMA hydrogel. The formed hydrogels were immersed in PBS and incubated on a shaker at 37 °C with a shaking speed of 50 rpm for 24 h. Subsequently, fluorescence imaging was performed using a CRI Maestro system (USA). To investigate the optimal *in vivo* concentration of C-VEGFMP, the peptide was incorporated into GelMA hydrogels at concentrations of 0.5 µM, 1 µM, 2 µM and 4 µM. These hydrogels were subsequently implanted into the dorsal muscle of rats. After 7 days, tissues surrounding the hydrogels were harvested and subjected to immunofluorescence staining for CD31 to evaluate the angiogenic capacity. All animal experiments were approved by the Experimental Animal Welfare and Ethics Committee, School of Public Health, Jilin University (SY: 2026-06-006).

### 2.3 Synthesis and characterization of ZIF-8@CuO/C-VEGFMP hydrogel

Based on previous research findings, the optimal loading concentration of ZIF-8@CuO in GelMA hydrogel was determined to be 0.15 wt%.^25^ Based on the *in vivo* angiogenesis results, 2 µM was selected as the working concentration of C-VEGFMP incorporated into the GelMA hydrogel. The surface morphologies of the hydrogels were observed by scanning electron microscopy (SEM, Zeiss EVO 18, Germany). The tensile strength of different hydrogels was measured using a material testing machine (68TM-5, Instron, UK). The clotting ability of the hydrogel was evaluated by the blood clotting index (BCI) assay. To test the photothermal properties of different hydrogels, an 808 nm near-infrared laser was used to irradiate the hydrogel samples under the following conditions: (1) four types of hydrogels (GelMA, ZIF-8@CuO, C-VEGFMP, and ZIF-8@CuO/C-VEGFMP) were irradiated at a power density of 1.0 W cm^−2^; (2) the photothermal response of the ZIF-8@CuO/C-VEGFMP hydrogel was evaluated at different power densities (0.25, 0.5, and 1.0 W cm^−2^); and (3) under the same irradiation conditions, the photothermal stability of the ZIF-8@CuO/C-VEGFMP hydrogel was evaluated over multiple heating–cooling cycles. ZIF-8@CuO-incorporated GelMA hydrogel samples (200 mg each) were immersed in 2 mL of water. Inductively coupled plasma–mass spectrometry (ICP-MS, NexION 1000G, PerkinElmer, USA) was employed to quantify the concentrations of Zn^2+^ and Cu^2+^ in the release medium after 1, 3, and 7 days of incubation. Circular hydrogel samples (diameter = 15 mm, thickness = 10 mm) from different groups were prepared, lyophilized and weighed to record initial dry weight (*W*_0_). Hydrogels were immersed in 15 mL of collagenase type II solution (5 U mL^−1^ in pH 7.4 PBS) and incubated at 37 °C on a shaker at 80 rpm. The collagenase-containing medium was refreshed every day to maintain enzyme activity. At predetermined time points (5, 10, 20 and 25 h), hydrogel samples were collected, rinsed with PBS, lyophilized, and weighed to obtain the residual dry weight (*W*_t_). The degradation rate was calculated as:
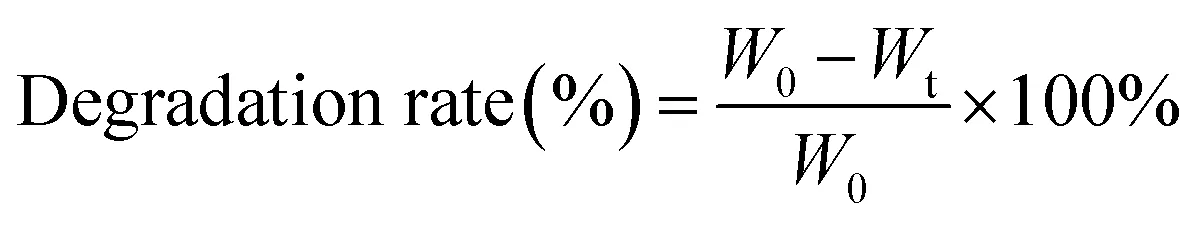


### 2.4 Determination of antibacterial activity

*Staphylococcus aureus* (*S. aureus*, ATCC 25923) and *Escherichia coli* (*E. coli*, ATCC 25922) were used as typical strains to evaluate the antibacterial performance of the hydrogels. Hydrogel samples (100 mg) were immersed in 1 mL of bacterial suspension (1 × 10^6^ CFU mL^−1^). For the ZIF-8@CuO/C-VEGFMP-NIR group, samples were immediately subjected to irradiation with an 808 nm NIR laser (1.0 W cm^−2^, 5 min). All groups were then incubated at 37 °C with shaking at 50 rpm for 24 h. Then, 100 µL bacterial suspension was spread onto agar plates and incubated for another 24 h. Finally, bacterial colonies on the agar plates were counted. The supernatant of each group was collected to detect OD_600_ values using a microplate reader (M200 Tecan, Switzerland). The inhibition rate was calculated by the following formula:
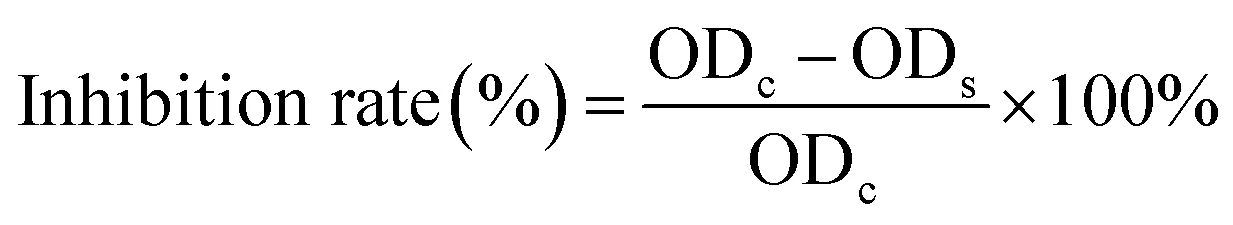
where OD_c_ represents the OD_600_ value of the bacterial suspension without hydrogel treatment, and OD_s_ represents the OD_600_ value of the bacterial supernatant co-cultured with composite hydrogels. The bacterial morphology was observed by SEM. To further evaluate the *in vivo* antibacterial efficacy, a full-thickness wound model was established in rats. A 10 mm-diameter, full-thickness wound was created on the dorsum of rats. A 10 µL aliquot of a mixed suspension of *E. coli* and *S. aureus* (each at 1 × 10^6^ CFU mL^−1^) was dropped onto the wound, followed by coverage with hydrogels from different groups. After 3 days, the tissues surrounding the wounds were harvested and subjected to Gram staining to evaluate the *in vivo* antibacterial activity of the materials.

### 2.5 Determination of anti-inflammatory ability

To evaluate the free radical scavenging ability of the hydrogels, a DPPH radical scavenging assay and dihydroethidium (DHE) tissue staining were employed. For the *in vivo* DHE staining, a subcutaneous pocket was created on the dorsum of rats, and hydrogels from different groups were implanted. After 24 h, the subcutaneous tissues were harvested and prepared as frozen sections. The tissue sections were incubated with DHE probe at room temperature for 1 h and observed under a fluorescence microscope (E80i, Nikon, Japan). For the *in vitro* DPPH assay, hydrogel extract was mixed with 100 µL of DPPH solution and incubated at room temperature in the dark for 30 min. The absorbance at 517 nm was recorded using a microplate reader (M200 Tecan, Switzerland). The DPPH scavenging rate was calculated according to the following formula:
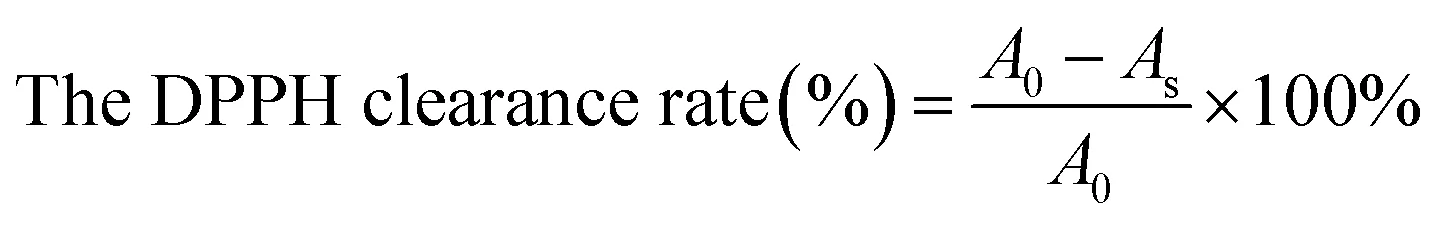
where *A*_0_ is the absorbance of pure DPPH solution, and *A*_s_ refers to the absorbance of DPPH solution mixed with hydrogel extracts. At the cellular level, NIH/3T3 cells were seeded at a density of 3 × 10^4^ cells per well in 12-well plates for 24 h. The culture medium was then replaced with fresh medium containing H_2_O_2_ (300 µM), and the hydrogel was added for co-culture for 24 h. Live and dead cells were detected by Calcein AM/PI double staining.

### 2.6 *In vitro* cell experiments

NIH/3T3 cells were co-cultured with different hydrogels in 24-well plates (3 × 10^4^ cells per well). On days 1, 3, and 7, the proliferation status of cells in different experimental groups was measured using the CCK-8 assay. On day 3, cells were stained with Calcein AM, respectively. The adhesion of cells on the surfaces of different hydrogels was observed using a fluorescence microscope. On day 7, cells were fixed with 4% paraformaldehyde and permeabilized with 0.1% Triton X-100, followed by incubation with primary antibodies against COL-1 (Abcam, ab34710) and CD31 (Abcam, ab182981), and then with FITC-labeled secondary antibody. The cytoskeleton and nuclei were counterstained with phalloidin and DAPI, respectively, and the cells were imaged under the same fluorescence microscope. On day 7, total RNA was extracted from the cells using TRIzol reagent (Invitrogen, USA), and it was reverse-transcribed into cDNA using a reverse transcription kit (Toyobo, Osaka, Japan). Quantitative analysis of the gene expression levels of type I collagen (COL-1) and CD31 was performed using a real-time PCR system (CFX96 Touch, Bio-Rad, USA), with glyceraldehyde-3-phosphate dehydrogenase (GAPDH) as the housekeeping gene for data normalization. The primer sequences are listed in Table S1.

The RAW264.7 cells were co-cultured with hydrogels from different groups and stimulated with lipopolysaccharide (LPS) for 24 hours. The expression levels of arginase-1 (Arg-1), interleukin-6 (IL-6), tumor necrosis factor-α (TNF-α), and interleukin-10 (IL-10) were determined using the same qPCR procedure as described above.

### 2.7 *In vivo* wound-healing assays

This study was approved by the Ethics Committee of Jilin University (SY:2026-06-006). Male Sprague-Dawley (SD) rats (8 weeks old) were divided into five groups: GelMA, ZIF-8@CuO, C-VEGFMP, ZIF-8@CuO/C-VEGFMP, and ZIF-8@CuO/C-VEGFMP-NIR. The hair on the dorsum of each rat was removed, and a circular full-thickness wound (10 mm in diameter) was created on the skin. Subsequently, a 10 µL aliquot of a mixed suspension of *E. coli* and *S. aureus* (each at 1 × 10^6^ CFU mL^−1^) was added to each wound. Hydrogels from different groups were then applied to the wounds. The ZIF-8@CuO/C-VEGFMP-NIR group was irradiated with an 808 nm NIR laser (1.0 W cm^−2^, 5 min) once every two days for one week. On days 1, 3, 5, 7, and 11, the wounds were photographed, and the wound area was measured using ImageJ software. The wound closure rate was calculated using the following formula:
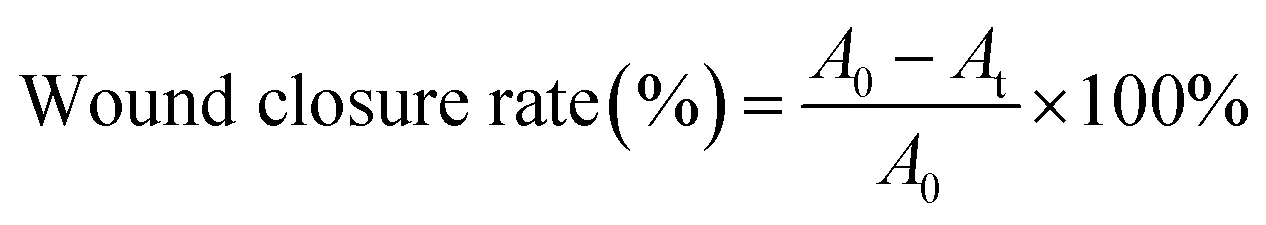
where *A*_0_ and *A*_t_ represent the initial area of the wound and the area of the wound at the time of measurement.

### 2.8 Histopathological assay

After 11 days of treatment, the rats were sacrificed, and the newly formed skin tissues were harvested and fixed with 4% paraformaldehyde. The tissues were then cryosectioned. The sections were stained with Masson's trichrome and hematoxylin and eosin (H&E), and images were captured under an upright microscope (BX53, Olympus, Japan). Immunofluorescence staining was performed to detect TNF-α, IL-10, and COL-1 (all antibodies from Abcam). The samples were observed and imaged under an upright fluorescence microscope (Axio Imager.M2, Zeiss, Germany).

### 2.9 Biosafety evaluation

On day 11 after wound repair, the heart, liver, spleen, lungs, and kidneys were harvested from the rats for H&E staining to evaluate the organ toxicity of the implanted hydrogels. In a separate biosafety experiment, serum samples were collected on day 21 after subcutaneous implantation and subjected to biochemical tests using an automatic chemistry analyzer (BS-240 Vet, Mindray, Shenzhen, China) to assess the *in vivo* biosafety of the materials.

### 2.10 Transcriptomic profiling

To further investigate the molecular mechanisms underlying the ZIF-8@CuO/C-VEGFMP-NIR treatment in the repair of infected skin wounds in rats, transcriptomic analysis was conducted on skin tissues isolated from the wound sites. The GelMA group served as the control, and the ZIF-8@CuO/C-VEGFMP-NIR group served as the experimental group. Total RNA was extracted from the tissue samples using TRIzol reagent and sent to BGI Tech Solutions Co., Ltd (BGI Tech) for RNA-seq. The sequencing data were analyzed using the Dr Tom multi-omics mining platform (BGI).

### 2.11 Statistical analysis

All data were analyzed using Origin 8.0 software (OriginLab Corporation, USA), and the results were expressed as the mean ± standard deviation (SD). The intergroup differences were analyzed by one-way analysis of variance (ANOVA). A *p*-value <0.05 was considered statistically significant.

## 3 Results and discussion

### 3.1 Characterization of ZIF-8@CuO and C-VEGFMP

In this study, we modified the biomimetic scaffold materials with ZIF-8@CuO and C-VEGFMP to enhance their various biological properties. First, we successfully synthesized ZIF-8@CuO nanoparticles. The TEM results (Fig. 1A) showed that the CuO nanoparticles were spherical, while the ZIF-8 nanoparticles exhibited a rhombic dodecahedral structure. Small particles were observed both within and on the surface of the ZIF-8 matrix, indicating that CuO nanoparticles were embedded in ZIF-8. The energy-dispersive X-ray spectroscopy (EDS) spectra (Fig. 1B and C) showed that the ZIF-8@CuO nanoparticles contained three elements: C, Zn, and Cu. The XRD patterns (Fig. 1D) showed that CuO exhibited characteristic peaks at 32.40°, 35.46°, 38.78°, 48.78°, 53.47°, and 58.25°. ZIF-8 exhibited peak positions at 7.33°, 10.40°, 12.69°, 18.02°, 22.14°, 24.49°, and 26.68°. The XRD diffraction peaks of ZIF-8@CuO were nearly identical to those of ZIF-8, indicating that the introduction of CuO did not change its crystal structure. The UV-vis spectrum (Fig. 1E) showed that ZIF-8 did not exhibit significant absorption from 700 to 1000 nm, while CuO and ZIF-8@CuO both showed broad absorption bands, indicating that ZIF-8@CuO had been successfully prepared. The Fourier transform infrared (FTIR) spectrum (Fig. 1F) showed that the characteristic peak at 593 cm^−1^ of CuO corresponded to the stretching vibration of the Cu–O bond. The FTIR spectrum of ZIF-8@CuO had similar characteristic peaks to ZIF-8, but there was a peak at 593 cm^−1^, which was caused by the stretching vibration of the Cu–O bond. As shown in Fig. S1A, the Raman spectra revealed that ZIF-8@CuO composites retained all characteristic Raman scattering peaks of pure ZIF-8 without obvious peak shifts, indicating that CuO modification did not destroy the imidazole ring skeleton and Zn–N coordination structure of ZIF-8. Compared with pure ZIF-8, ZIF-8@CuO exhibited a significantly decreased overall Raman scattering intensity, which was attributed to the light extinction and absorption effects of surface-loaded CuO. TGA measurements (Fig. S1B) were conducted to explore the thermal degradation behaviors of ZIF-8 and ZIF-8@CuO. Both samples displayed slight weight loss at 25–200 °C, corresponding to the removal of surface adsorbed water and residual solvents. In the range of 200–480 °C, both materials exhibited excellent thermal stability, while ZIF-8@CuO exhibited a higher residual mass, demonstrating that the incorporation of CuO effectively improved the medium-temperature thermal stability of the ZIF-8 framework.

**Fig. 1:**
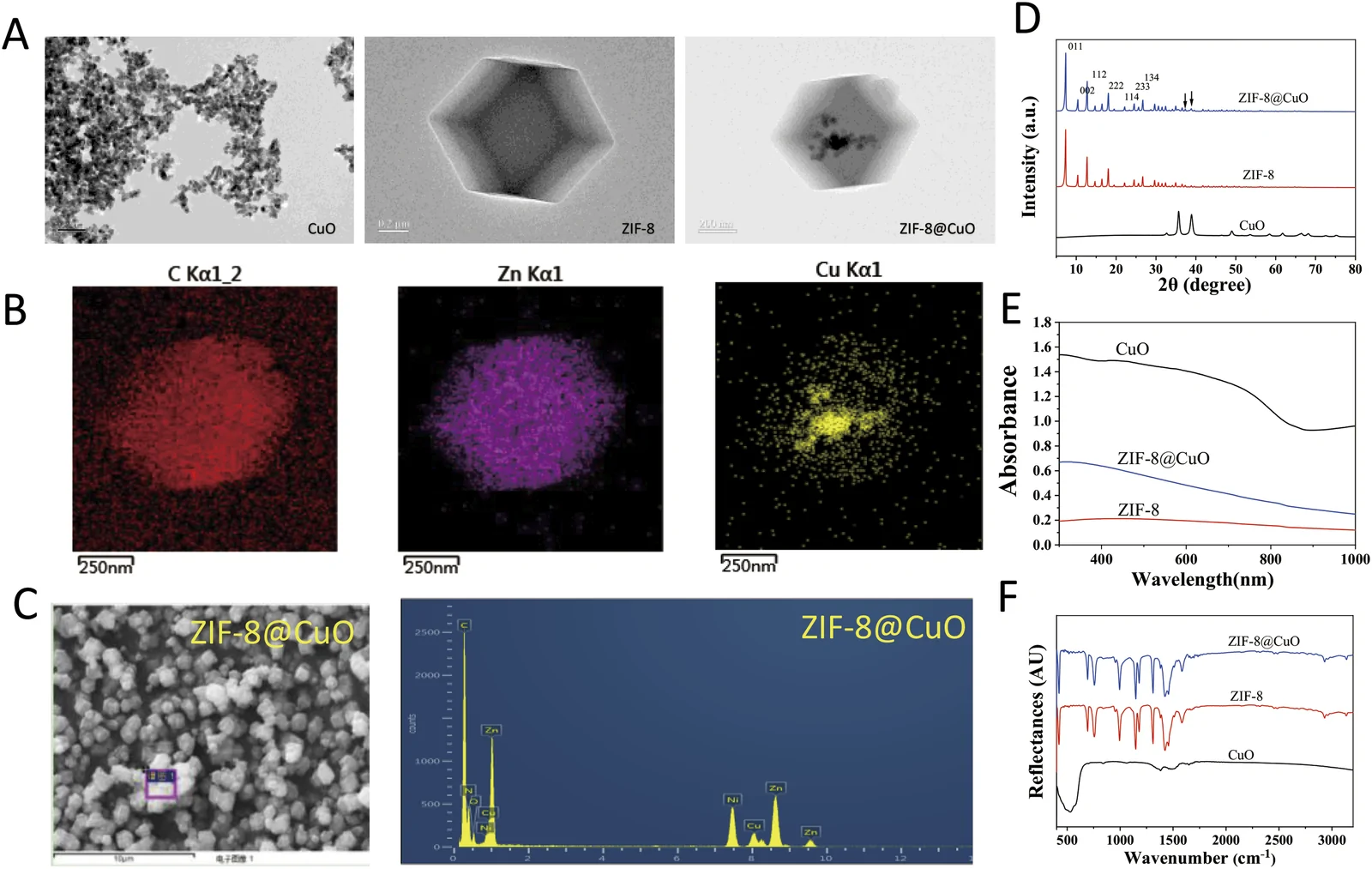
(A) TEM images of CuO, ZIF-8 and ZIF-8@CuO nanoparticles. Bar lengths are 200 nm. (B) Mapping results of ZIF-8@CuO nanoparticles. Bar lengths are 250 nm. (C) SEM images and EDS results of ZIF-8@CuO nanoparticles. Bar lengths are 10 µm. (D) XRD, (E) UV-vis spectrum and (F) FTIR results of CuO, ZIF-8 and ZIF-8@CuO nanoparticles.

In this study, we introduced the collagen-binding domain (CBD) sequence (TKKTLRT) into VEGFMP to generate a modified peptide with enhanced material-binding ability. First, the molecular weights (MW) of VEGFMP and C-VEGFMP were characterized by electrospray ionization mass spectrometry (ESI-MS) (Fig. S2 and S4). The ESI-MS results showed that the average MW of VEGFMP is 1910.73 Da and that of C-VEGFMP is 2739.63 Da, both in good agreement with their theoretical values, confirming the successful synthesis of both peptides. As shown in Fig. S3 and S5, HPLC analysis confirmed that both VEGFMP and C-VEGFMP were >95% pure. Subsequently, peptide binding assays were performed by incorporating FITC-labeled VEGFMP or C-VEGFMP into GelMA hydrogels and imaging the fluorescence retention after washing. The fluorescence images showed that the fluorescence intensity in the C-VEGFMP group was significantly higher than that in the VEGFMP group, whereas no fluorescence signal was detected in the control group. Quantification of the average fluorescence intensity revealed that the C-VEGFMP group exhibited approximately 2.65-fold higher signal intensity than the VEGFMP group. These results demonstrated that C-VEGFMP has strong binding retention in GelMA hydrogels.

To determine the optimal concentration of C-VEGFMP for promoting angiogenesis, C-VEGFMP-loaded GelMA hydrogels at different concentrations (0.5, 1, 2, and 4 µM) were subcutaneously implanted in rats, and neovascularization was evaluated by CD31 immunofluorescence staining (Fig. 2B and C). The results showed that treatment with 2 µM C-VEGFMP significantly increased the number of new blood vessels in the implanted area. However, when the C-VEGFMP concentration was further increased, the pro-angiogenic effect plateaued. Therefore, 2 µM was selected as the optimal concentration for C-VEGFMP loading in this study.

**Fig. 2:**
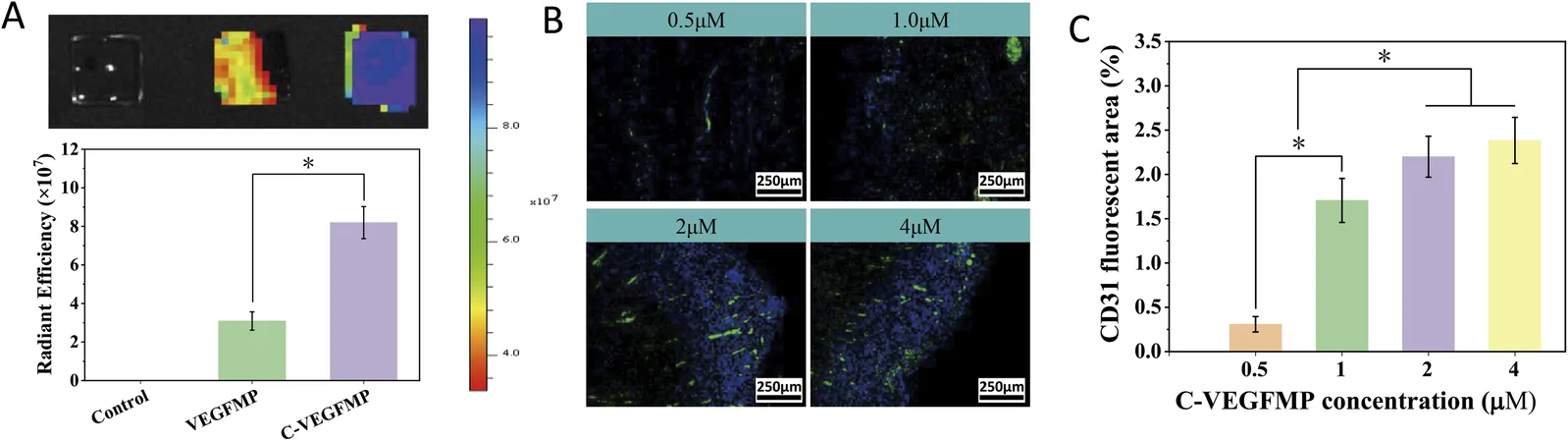
(A) The binding ability of peptide to GelMA hydrogel. (B) Immunofluorescent images and (C) quantitative analysis for CD31 expression. Scale bar: 250 µm. *p < 0.05, *n* = 3.

### 3.2 Characterization of ZIF-8@CuO/C-VEGFMP hydrogel

After the successful preparation of ZIF-8@CuO and C-VEGFMP, both components were incorporated into GelMA hydrogel to fabricate a multifunctional skin repair material. First, the general morphology of the ZIF-8@CuO/C-VEGFMP hydrogel was observed. As shown in Fig. 3B, the pre-gel solution of ZIF-8@CuO/C-VEGFMP was a black viscous liquid that transformed into a gel upon exposure to 405 nm violet light. SEM images indicated that all hydrogels possessed a typical interconnected porous structure, which is favorable for nutrient and waste exchange (Fig. 3A). The pores of the GelMA and C-VEGFMP hydrogels had relatively smooth surfaces, while the addition of ZIF-8@CuO significantly increased the roughness of the pore surface, and some ZIF-8@CuO particle agglomerates were found on the pore surface. Such interconnected porous architecture is essential for mass transport within the hydrogel matrix, and a similar structural–performance relationship has also been reported in porous composite hydrogel systems.^26^ As shown in Fig. 3C, the FTIR results showed that the GelMA hydrogel exhibited characteristic amide I and amide II absorption peaks at 1650 cm^−1^ and 1540 cm^−1^, confirming the presence of the gelatin matrix and successful methacrylation. Compared with the GelMA hydrogel, the FTIR spectra of the ZIF-8@CuO, C-VEGFMP, and ZIF-8@CuO/C-VEGFMP groups did not show significant changes, which might be due to the relatively small loading amount of ZIF-8@CuO and C-VEGFMP. As shown in Fig. 3D, the BCI value of the GelMA group was 0.65 ± 0.071. In the C-VEGFMP group, the BCI value showed no significant change. After the incorporation of ZIF-8@CuO, the hydrogel's ability to promote RBC and platelet aggregation slightly improved, and the BCI value decreased to 0.47 ± 0.114. The hemostatic performance of ZIF-8@CuO/C-VEGFMP hydrogel in diffuse hemorrhagic wounds was also evaluated using a rat tail injury model. As shown in Fig. 3E, the ZIF-8@CuO and ZIF-8@CuO/C-VEGFMP groups effectively controlled rat tail bleeding. Previous studies have found that the released Zn^2+^ from ZIF-8 can simulate platelet Zn^2+^ secretion, positively amplifying the coagulation signal.^27,28^ Additionally, Cu^2+^ also promotes the formation of a strong fibrin network and facilitates platelet adhesion and aggregation.^29,30^ As shown in Fig. 3F, S6, and Table S2, different hydrogels were assessed in terms of blood compatibility, internal organ toxicity and serum biochemistry. All hydrogel materials exhibited hemolysis rates below 5%, without inducing organ toxicity in rats. Moreover, all serum biochemical parameters were within normal ranges. These results collectively confirm the outstanding biocompatibility and *in vivo* biosafety of the ZIF-8@CuO/C-VEGFMP hydrogel. The swelling results of different hydrogels showed that the swelling rate of the ZIF-8@CuO group was slightly lower than that of the GelMA group (Fig. 3G). This might be attributed to the physical filling of the hydrogel pores by ZIF-8@CuO, which reduces the free volume available for water molecules to enter.^31^ However, notably, the ZIF-8@CuO/C-VEGFMP hydrogel still maintained a relatively high water-absorption capacity, with the equilibrium swelling ratio remaining above 160%, indicating that the addition of ZIF-8@CuO did not disrupt the hydrophilic network of GelMA.

**Fig. 3:**
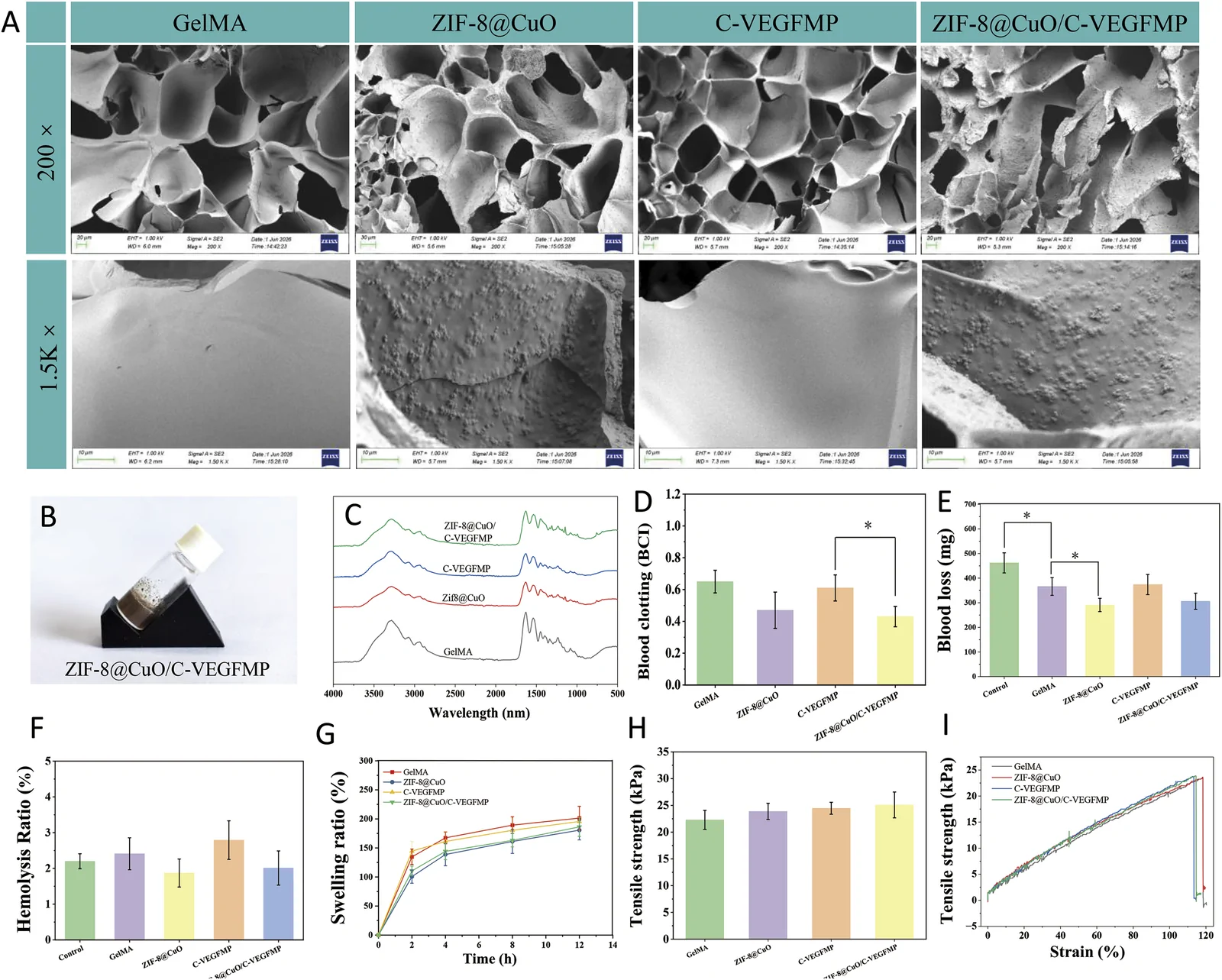
(A) SEM images of different hydrogels. Scale bar: 30 µm (upper) and 10 µm (under). (B) The macroscopic images of ZIF-8@CuO/C-VEGFMP hydrogels. (C) FTIR spectra of different hydrogels. (D) BCI values of different hydrogels, and (E) blood loss in a rat tail injury model. (F) Hemolysis, (G) swelling ratio and (H and I) mechanical tensile results of different hydrogels. *p < 0.05, *n* = 3.

To further clarify the contributions of each functional component and ion-mediated biological effects within the composite hydrogel, ICP-MS was used to monitor the release behavior of Cu^2+^ and Zn^2+^ from the ZIF-8@CuO-incorporated hydrogel over 7 days of incubation. As shown in Fig. S7, the concentrations of Cu^2+^ and Zn^2+^ continuously increased with incubation time, exhibiting a sustained release profile without obvious burst release. Zn^2+^ originates from the gradual degradation of the ZIF-8 framework, while Cu^2+^ is released *via* the slow dissociation of the CuO component. This sustained ion release enables a stable local metal-ion microenvironment: Zn^2+^ participates in regulating coagulation signals and promoting cell proliferation, whereas Cu^2+^ endows the material with antibacterial, antioxidant and pro-angiogenic activities. Meanwhile, the continuously released Zn^2+^ and Cu^2+^ can form coordination interactions with polar groups on GelMA polymer chains, which regulate the network structure and mechanical properties of hydrogels. These ion release results demonstrated the ion supply pattern of ZIF-8@CuO nanoparticles embedded in the hydrogel matrix, and provided direct experimental evidence for understanding the synergistic hemostasis, antibacterial and tissue repair mechanisms of this multifunctional hydrogel.

Fig. S8 presents the *in vitro* enzymatic degradation behavior of four hydrogel groups upon exposure to type II collagenase. Overall, the degradation rates of all hydrogels gradually increased with extended incubation time, exhibiting time-dependent enzymatic degradation characteristics. The C-VEGFMP group exhibited the fastest degradation rate and the highest degradation percentage at all time points, followed by the GelMA group. The ZIF-8@CuO group degraded more slowly than the GelMA group. The ZIF-8@CuO/C-VEGFMP composite hydrogel exhibited the lowest degradation rate across all time points, showing the slowest degradation kinetics. These results demonstrated that incorporation of the C-VEGFMP peptide alone accelerated the enzymatic degradation of GelMA hydrogels, whereas ZIF-8@CuO nanoparticles effectively retarded the degradation of the hydrogel. When ZIF-8@CuO and C-VEGFMP were co-incorporated into the hydrogel system, metal ions released from ZIF-8@CuO could form additional coordination crosslinks with both the peptides and GelMA polymer chains, further improving network stability and ultimately endowing the composite hydrogel with enhanced resistance to enzymatic degradation.

Fig. 3H and I show the tensile strength and stress–strain curves of hydrogels from different groups. Mechanical testing shows that the tensile strength of ZIF-8@CuO, C-VEGFMP, and ZIF-8@CuO/C-VEGFMP hydrogels was slightly higher than that of the GelMA group. The Zn^2+^ and Cu^2+^ released from the ZIF-8 and CuO can form coordination bonds with the carboxyl (–COOH) and amino (–NH_2_) groups on the GelMA chains, significantly increasing the network crosslinking density and endowing the hydrogel with stronger resistance to deformation.^32^ It is hypothesized that the collagen-binding domain of C-VEGFMP specifically binds to GelMA, thereby increasing the crosslinking density of GelMA and moderately enhancing the tensile strength of the hydrogel.^33^ Improved mechanical properties are highly desirable for wound dressings to withstand skin movement, which has been emphasized in a recent review on mechanically active wound dressings.^34^

### 3.3 Photothermal and antibacterial performance of ZIF-8@CuO/C-VEGFMP hydrogel

Give these favorable physicochemical properties, we next evaluated the photothermal and antibacterial performance of the hydrogels. In this study, the photothermal properties of different hydrogels were investigated under near-infrared irradiation (NIR). As shown in the thermal imaging (Fig. 4A and B), after 300 s of irradiation, no significant heating areas were observed in the GelMA and C-VEGFMP groups; while the ZIF-8@CuO and ZIF-8@CuO/C-VEGFMP groups exhibited pronounced hotspots, with the temperature rising to approximately 42 °C. Additionally, irradiation experiments were conducted on the hydrogels at various power densities (0.25, 0.5, 1.0 W cm^−2^). As the power density increased, the temperature elevation increased (Fig. 4C), indicating that different power densities could modulate the photothermal effect of the ZIF-8@CuO/C-VEGFMP hydrogel. The photothermal stability test after three on/off cycles showed that the temperature profiles were reproducible, confirming that the photothermal properties of the ZIF-8@CuO/C-VEGFMP hydrogel exhibit good photothermal stability (Fig. 4D). CuO exhibits excellent near-infrared response properties, which is attributed to its unique optical characteristics, allowing it to absorb light ranging from ultraviolet to near-infrared.^35^

**Fig. 4:**
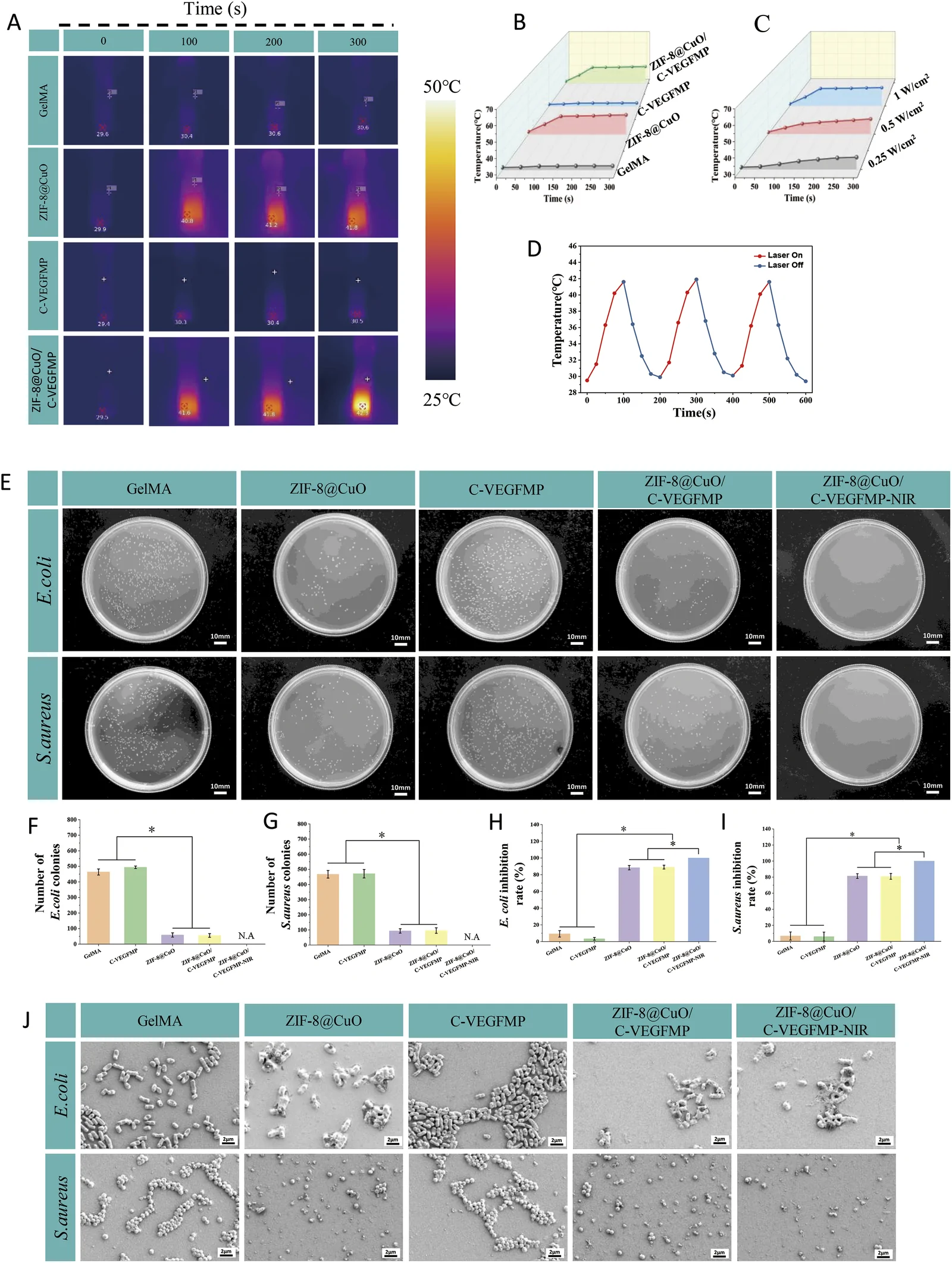
(A) Thermal images of different hydrogels irradiated by NIR (1 W cm^−2^) for different times. (B) Corresponding temperature changes of different hydrogels irradiated by NIR (1 W cm^−2^). (C) Temperature changes of ZIF-8@CuO/C-VEGFMP hydrogels under NIR irradiation at various power densities. (D) Photothermal stability of the ZIF-8@CuO/C-VEGFMP hydrogels. (E) Results of the spread plate method; scale bar: 10 mm. The results of the bacterial colony counts and relative inhibitory rates of *E. coli* (F and H) and *S. aureus* (G and I) in each experimental group. (J) SEM images of bacterial morphology, scale bar: 2 µm. *p < 0.05, *n* = 3.

Subsequently, the antibacterial performance of different hydrogels was tested *via* the plate counting method combined with bacterial morphological observation. The results of the plate spreading assay (Fig. 4E–G) revealed that the colony counts of the GelMA and C-VEGFMP groups ranged from 467 to 484, while the colony counts of the ZIF-8@CuO group decreased to 59–96. No detectable bacterial colonies were on the agar plates for the ZIF-8@CuO/C-VEGFMP-NIR group. As shown in Fig. 4H and I, the ZIF-8@CuO/C-VEGFMP-NIR group achieved OD_600_-measured antibacterial rates of 99.6 ± 2.71% against *E. coli* and 99.7 ± 3.96% against *S. aureus*, respectively. As shown in Fig. S9, Gram staining was performed to assess the *in vivo* antibacterial performance of hydrogels in infected rat wounds. At 3 days post-treatment, abundant bacteria (both Gram-positive and Gram-negative) were observed in the GelMA and C-VEGFMP groups, exhibiting high bacterial-positive area fractions. The ZIF-8@CuO group showed a moderate reduction in bacterial burden. The ZIF-8@CuO/C-VEGFMP group further reduced the bacterial burden, while the ZIF-8@CuO/C-VEGFMP-NIR group displayed the lowest bacterial positive area fraction with effective bacterial clearance. Significant differences were noted between groups (*p < 0.05, *n* = 3), demonstrating the superior *in vivo* antibacterial efficacy of this combined therapeutic system. These results demonstrated that the ZIF-8@CuO/C-VEGFMP hydrogel combined with photothermal therapy significantly inhibited bacterial growth. The bacterial morphology observed by SEM showed that in the GelMA and C-VEGFMP groups, *S. aureus* and *E. coli* had spherical and rod-shaped structures with intact and smooth surfaces; in contrast, the bacterial surfaces in the ZIF-8@CuO and ZIF-8@CuO/C-VEGFMP groups were rough, shrunken, and deformed (Fig. 4J). The ZIF-8@CuO/C-VEGFMP-NIR group exhibited more severe deformation and contraction, indicating an enhanced antibacterial effect. Under NIR irradiation, the antibacterial effect of ZIF-8@CuO/C-VEGFMP hydrogel relies on a multimodal synergistic mechanism. First, photothermal hyperthermia effect occurs when the doped CuO nanoparticles are exposed to 808 nm near-infrared light and efficiently convert light energy into localized heat (with the temperature reaching approximately 42 °C). Mild-to-moderate hyperthermia will increase the permeability of bacterial cell membranes and damage the integrity of proteins and enzymes, but will not cause extensive secondary thermal damage to the surrounding healthy tissues.^36^ Furthermore, ZIF-8@CuO can effectively inhibit bacterial growth through the synergistic effect of continuous release of Zn^2+^ and Cu^2+^.^25,37^ NIR stimulation significantly enhanced the antibacterial effect of the ZIF-8@CuO/C-VEGFMP hydrogel, which is attributed to the excellent photothermal conversion performance of the ZIF-8@CuO/C-VEGFMP hydrogel. Such a synergistic antimicrobial mode combining mild photothermal hyperthermia and metal-ion-mediated bactericidal action has been reviewed in a recent review on temperature-mediated antimicrobial strategies.^38^ Under NIR irradiation, the composite hydrogel exhibited obvious biofilm-disrupting capacity, which is consistent with an earlier report on near-infrared-activated nanoplatform for infected wound therapy.^39^

### 3.4 Anti-inflammatory properties of ZIF-8@CuO/C-VEGFMP hydrogel

Since infected wounds are also characterized by oxidative stress and inflammation, we next assessed the anti-inflammatory properties of the hydrogel. To test the antioxidant ability of ZIF-8@CuO/C-VEGFMP hydrogel, live/dead staining and tissue DHE staining were used to observe cell viability and reactive oxygen species (ROS) levels in the tissue under an oxidative stress microenvironment. Firstly, H_2_O_2_ was added to the culture medium to mimic the oxidative stress microenvironment and the cells were co-cultured with different hydrogels. As shown in Fig. 5A, live/dead cell staining after 24 h of culture revealed that a large number of dead cells (stained red) were observed in the GelMA and C-VEGFMP groups. However, a greater number of live cells (stained green) were observed in the ZIF-8@CuO and ZIF-8@CuO/C-VEGFMP groups. The DHE staining of the tissues revealed that the control, GelMA and C-VEGFMP groups showed obvious red fluorescence, while the ZIF-8@CuO and ZIF-8@CuO/C-VEGFMP groups displayed only weak red fluorescence. Quantitative analysis of DHE fluorescence intensity (Fig. 5B) confirmed these observations. In the early stage of skin wound repair, the predominant macrophage phenotype is the M1 phenotype, and they secrete pro-inflammatory factors such as TNF-α and IL-6. When these cells undergo M2 polarization, they transform the wound immune microenvironment into an “anti-inflammatory repair” phenotype.^40^ To investigate the effects of different hydrogels on macrophage polarization, we established an *in vitro* inflammatory model using RAW264.7 macrophages stimulated with LPS and co-cultured them with different hydrogels. The expression levels of M2 -related factors (Arg-1, IL-10) and M1-related inflammatory factors (TNF-α, IL-6) further demonstrated that the ZIF-8@CuO/C-VEGFMP hydrogel most effectively induced M2 macrophage polarization and could reverse M1 macrophage polarization in an inflammatory environment (Fig. 5C).

**Fig. 5:**
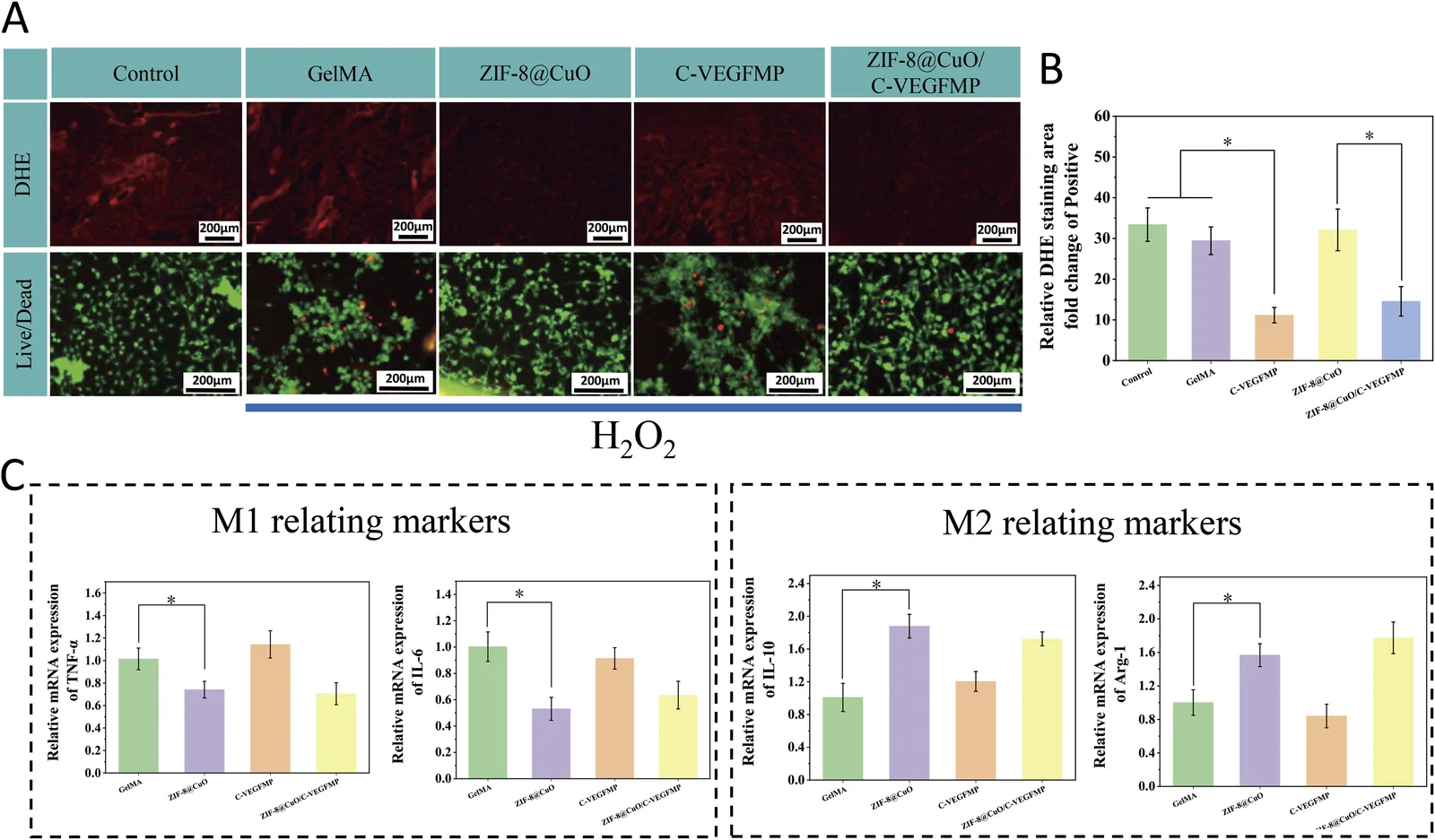
(A) Live/dead cell staining after coculture with H_2_O_2_, and ROS scavenging of different hydrogels. Scale bar: 200 µm. (B) Mean fluorescence intensity of ROS in different hydrogels. (C) The expression of TNF-α, IL-6, Arg-1 and IL-10 in macrophage co-cultured with different hydrogels. *p < 0.05, *n* = 3.

In the ZIF-8@CuO/C-VEGFMP hydrogel system, degrading ZIF-8 continuously releases Zn^2+^, which acts as a key mediator. It can reverse the excessive activation of LPS-induced M1 macrophages and promote their polarization toward the tissue-repair-oriented M2 phenotype. This phenotypic reprogramming is achieved through multiple signaling pathways, reactive oxygen species (ROS) clearance, mitochondrial homeostasis maintenance, and transcription factor reprogramming.

LPS stimulation activates the macrophage TLR4 receptor, initiates IKK-mediated IκBα degradation, allows NF-κB p65 nuclear translocation, and significantly upregulates the expression of M1 pro-inflammatory genes such as TNF-α, IL-6, and iNOS.^41^ Zn^2+^ can upregulate IκBα protein levels, inhibit the ubiquitination and degradation of IκBα, retain NF-κB p65 in the cytoplasm, and prevent its nuclear translocation; it can also upregulate the deubiquitinating enzyme A20, inhibiting upstream TLR4 inflammatory signaling.^42^ Additionally, Zn^2+^ reduces the phosphorylation of p38, ERK, and JNK, attenuating the MAPK pathway and thereby collectively inhibiting M1 polarization.^43^

While inhibiting M1 signaling, zinc ions also activate signaling pathways that promote the M2 phenotype. Moreover, Zn^2+^ enhances antioxidant defense, scavenges excess intracellular ROS, alleviates oxidative stress, and preserves mitochondrial integrity. The restoration of mitochondrial function is a prerequisite for the metabolic reprogramming of macrophages from M1 to M2. In the composite system of this study, CuO releases Cu^2+^, which acts synergistically with Zn^2+^ to significantly enhance the aforementioned immunomodulatory effects. Therefore, the ZIF-8@CuO/C-VEGFMP hydrogel can scavenge excess ROS in the microenvironment, alleviate cellular oxidative stress, preserve cell viability, and provide a favorable microenvironment for skin tissue regeneration.

### 3.5 *In vitro* cell tests

We then examined the cytocompatibility and pro-regenerative effects of the hydrogel *in vitro*. Using the CCK-8 assay, the effect of the ZIF-8@CuO/C-VEGFMP hydrogel on cell proliferation was investigated. As shown in Fig. 6C, the cell proliferation in the ZIF-8@CuO group was higher than that in the GelMA group, indicating that ZIF-8@CuO promoted cell proliferation. After the incorporation of C-VEGFMP, cell proliferation in each group further increased, indicating that this modification method of incorporating collagen-binding sequences does not compromise VEGFMP bioactivity. Live cell staining (Fig. 6A) showed that NIH/3T3 cells appeared as bright green, spindle-shaped cells. On day 3, statistical analysis of the cell area within the field of view showed that the C-VEGFMP group and the ZIF-8@CuO/C-VEGFMP group had the highest cell density, followed by the ZIF-8@CuO group, while the GelMA group exhibited the lowest cell density (Fig. 6F). The effects of ZIF-8@CuO/C-VEGFMP hydrogel on modulating COL-1 and CD31 expression were evaluated using qPCR and immunofluorescence staining. qPCR results (Fig. 6E and H) showed that both the ZIF-8@CuO group and the C-VEGFMP group exhibited upregulated expression of COL-1 and CD31 on day 7, with the ZIF-8@CuO/C-VEGFMP group demonstrating the highest upregulation trend of COL-1 and CD31 expression. As shown in Fig. 6B, in the ZIF-8@CuO/C-VEGFMP group, the immunofluorescence intensities of COL-1 and CD31 were the strongest, with the highest expression levels, and the ZIF-8@CuO and C-VEGFMP groups followed, while the GelMA group had the weakest expression. Quantitative analysis (Fig. 6D and G) further confirmed this trend. Studies have shown that Zn^2+^ can activate the cAMP-PKA signaling pathway, triggering the phosphorylation of extracellular signal-regulated kinase (ERK), and subsequently activating the mitogen-activated protein kinase (MAPK) signaling pathway.^44,45^ These activated signaling pathways can promote collagen secretion and tissue regeneration. Meanwhile, Cu^2+^ can inhibit the degradation of HIF-1α, mimic the hypoxic effect, and thereby activate the HIF-1 signaling pathway, promoting angiogenesis.^46^ VEGFMP binds to VEGFR on cell surfaces, activating the PI3K/Akt and MAPK/ERK pathways, improving local blood supply and nutrient supply, thereby stimulating fibroblast activation and enhancing collagen synthesis and deposition.^47,48^ These results indicated that the ZIF-8@CuO/C-VEGFMP hydrogel could effectively enhance cell viability, and upregulate the expression of genes related to tissue regeneration.

**Fig. 6:**
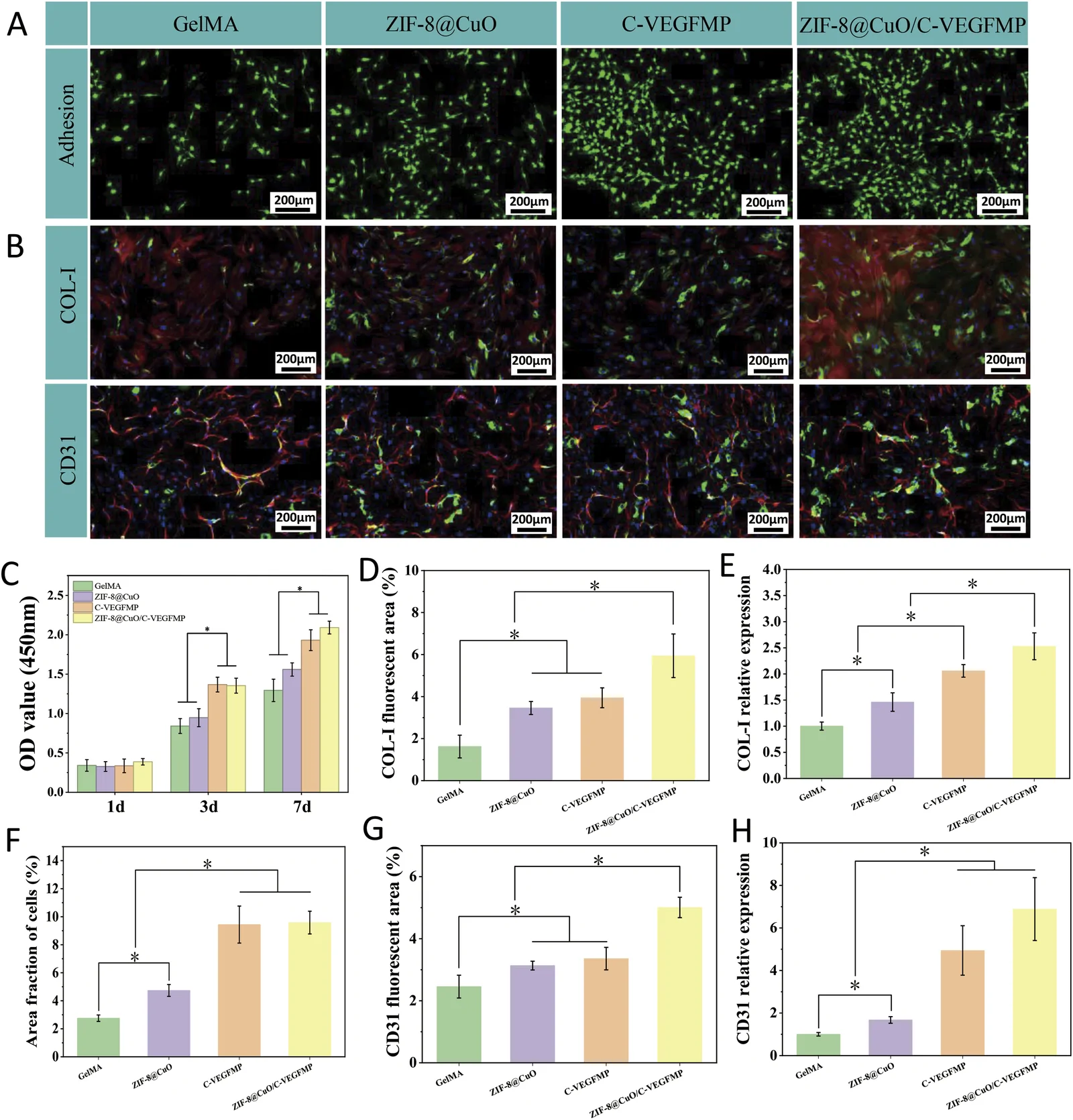
(A) The live cell staining image of NIH3T3 cells on different hydrogels surface. (B) COL-1 and CD31 immunofluorescence staining. (C) Quantitative analysis of biocompatibility of the hydrogels by CCK-8. (D and G) Quantitative analysis of the results of immunofluorescence staining results. (F) Cell area fraction of NIH3T3 cells cultured on different hydrogels. (E and H) The relative gene expression of COL-1 and CD31 respectively. Scale bar: 200 µm, *p < 0.05, *n* = 3.

### 3.6 Repair of infected skin wounds

To further evaluate the therapeutic effects of the hydrogel and photothermal therapy on infected skin wounds, a rat model of infected skin wound was established and treated with different treatments. The photothermal treatment group received short-term NIR irradiation during the first week. The hydrogels were replaced every two days, and the wounds were photographed. Fig. 7A shows representative wound photographs on days 0–11. Among all experimental groups, the GelMA group had the slowest wound healing rate. The wound closure rates of the ZIF-8@CuO and C-VEGFMP groups were higher than those of the GelMA group. After treatment with the ZIF-8@CuO/C-VEGFMP hydrogel, wound healing was further accelerated, demonstrating the synergistic effect of the two components in promoting wound healing (Fig. 7B). Among all the experimental groups, the ZIF-8@CuO/C-VEGFMP-NIR group had the fastest wound healing rate. As shown in Fig. 7C, after 11 days, the wounds of the ZIF-8@CuO/C-VEGFMP-NIR group were almost completely closed, and the wound closure rate was close to 100%. These findings suggest that ZIF-8@CuO can effectively enhance the antibacterial properties of the hydrogel, inhibit wound infection, and thereby create favorable conditions for rapid wound healing. Meanwhile, C-VEGFMP continuously exerts its pro-angiogenic function on the wound surface, promoting angiogenesis and collagen deposition. Under near-infrared light irradiation, the ZIF-8@CuO/C-VEGFMP hydrogel exhibits photothermal and photodynamic effects. This is attributed to CuO's ability to convert light energy into heat and produce reactive oxygen species (ROS).^49,50^ Combined with the continuous release of Zn^2+^ and Cu^2+^ from the ZIF-8@CuO, these effects act synergistically to achieve multimodal antibacterial activity, thereby effectively killing bacteria and promoting wound healing.

**Fig. 7:**
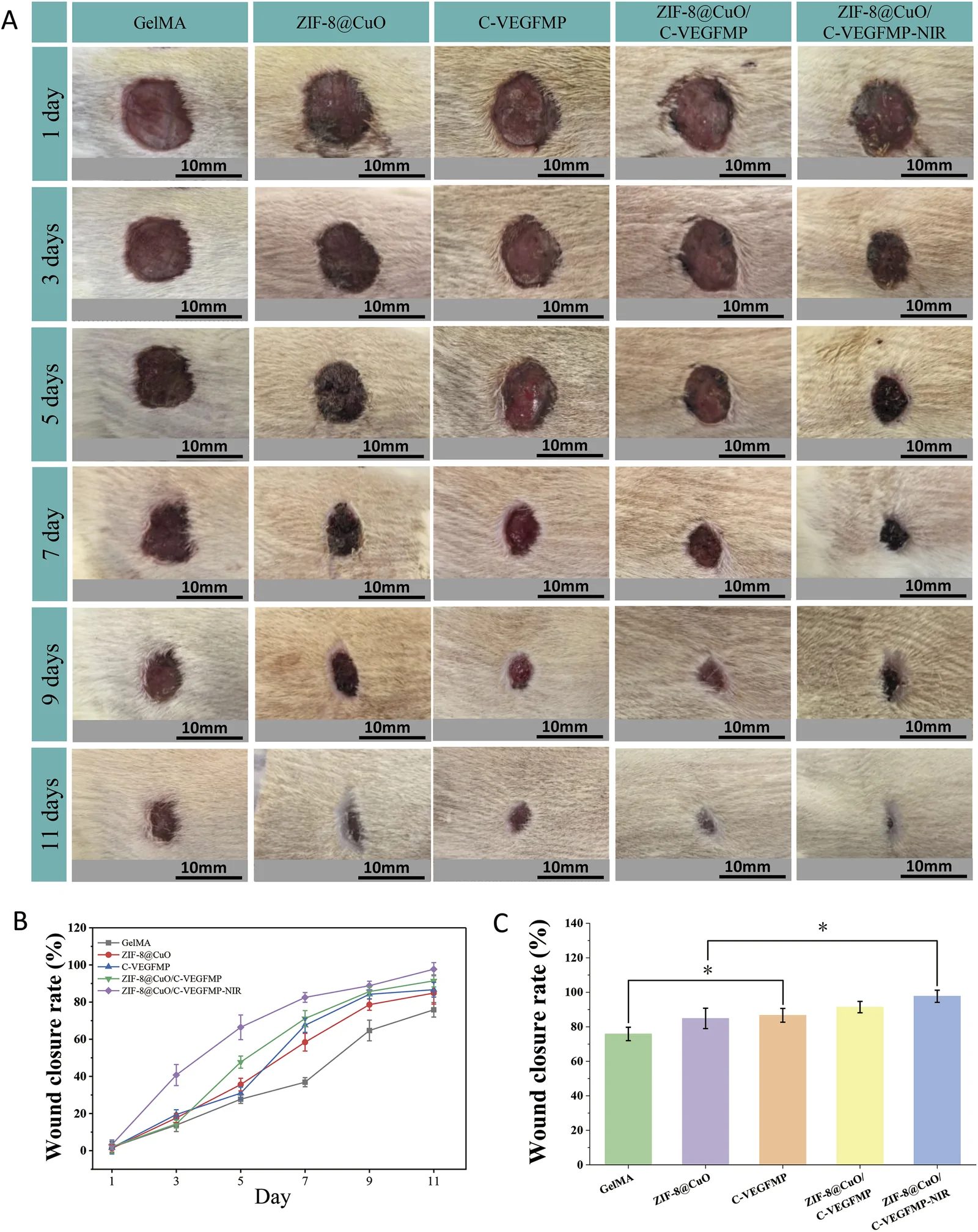
(A) Photographs of the skin wound. Scale bar:10 mm. (B) Quantitative statistical analysis of wounds closure rate. (C) The wound closure rate of each experimental group 11 days later. *p < 0.05, *n* = 3.

### 3.7 Histological analysis of skin repair

By histological and immunofluorescence analysis, the wound healing effects of different hydrogels and photothermal therapy were evaluated. H&E staining and Masson staining (Fig. 8A) showed that the epidermal layer of the wounds treated with ZIF-8@CuO/C-VEGFMP and ZIF-8@CuO/C-VEGFMP-NIR exhibited a more continuous epidermal layer with less scab formation, indicating that the rate of epithelial regeneration was accelerated. Masson staining further revealed that the collagen deposition (blue staining) in the ZIF-8@CuO/C-VEGFMP-NIR group was significantly increased compared with other groups, suggesting a stronger extracellular matrix remodeling capacity. Immunofluorescence staining (Fig. 8B) showed that the COL-I-positive area in the C-VEGFMP group was larger than that in the ZIF-8@CuO group. After the combined treatment of ZIF-8@CuO and C-VEGFMP, the positive area of COL-I further increased. Additionally, in the ZIF-8@CuO group, the expression of TNF-α (an inflammatory marker) was significantly reduced, while the expression of IL-10 (an anti-inflammatory marker) was increased, and this trend was not observed in the C-VEGFMP group, indicating that the anti-inflammatory components in the hydrogel were mainly derived from ZIF-8@CuO. The ZIF-8@CuO/C-VEGFMP-NIR group had the strongest COL-I expression among all groups, and TNF-α expression was significantly reduced, and the IL-10 expression was increased, suggesting that the transition from the inflammatory stage to the proliferative/remodeling stage of the wound healing process was accelerated. Quantitative analysis further confirmed these observations (Fig. 8C–E). These results indicated that the near-infrared light-activated ZIF-8@CuO/C-VEGFMP hydrogel effectively promoted collagen deposition, regulated the local immune microenvironment, and accelerated wound healing.

**Fig. 8:**
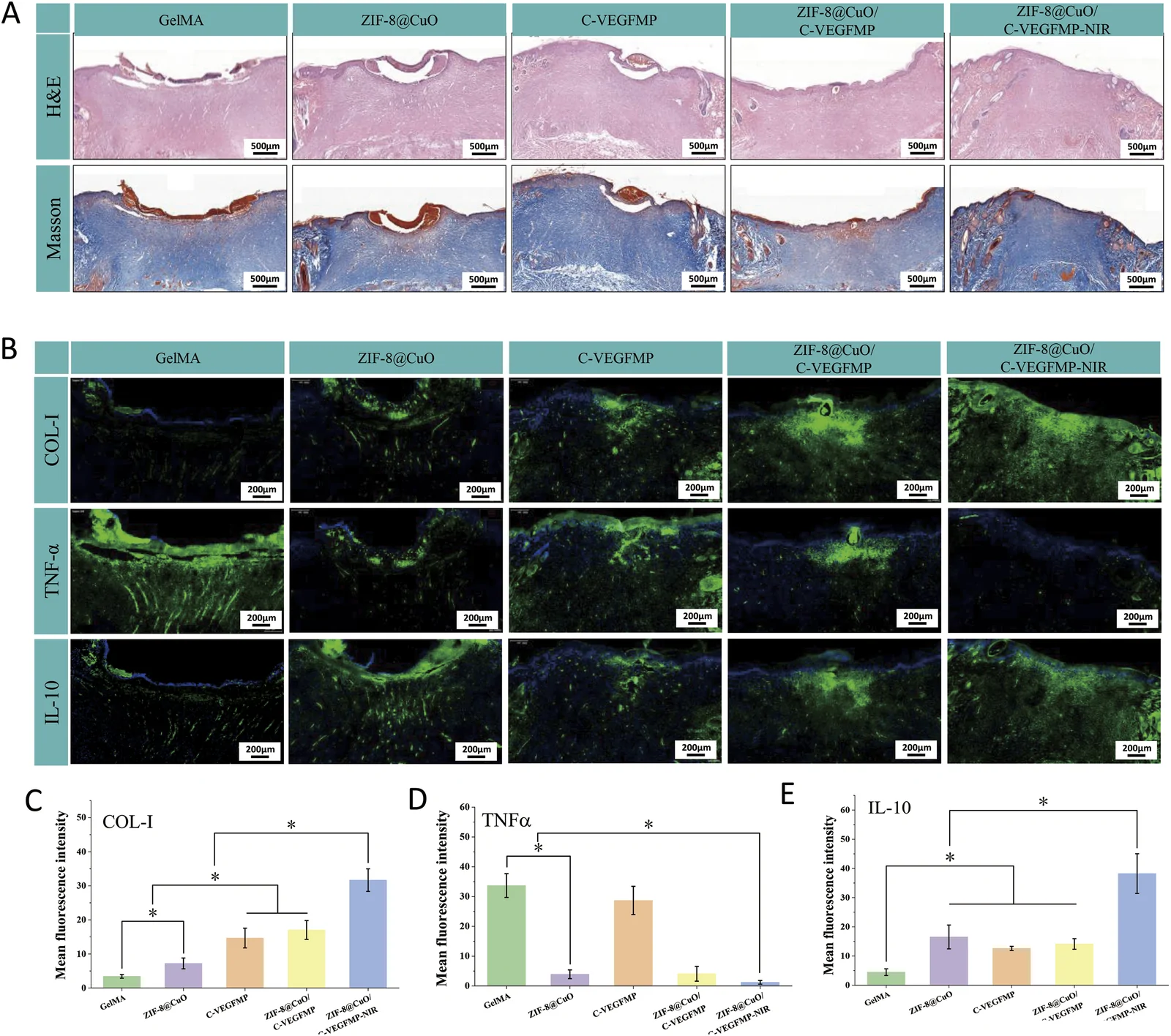
(A) HE and Masson staining of skin tissue, scale bar: 500 µm. (B) Immunofluorescence images and the quantitative statistical analysis of (C) COL-1, (D) TNF-α, and (E) IL-10 positive area, scale bar: 200 µm. *p < 0.05, *n* = 3.

### 3.8 Transcriptomic signatures

To explore the underlying molecular mechanisms, transcriptomic profiling was subsequently performed. As shown in Fig. S10, transcriptomic analysis revealed that 9 genes were significantly upregulated and 25 genes were significantly downregulated. The Gene IDs of upregulated and downregulated genes are listed in Tables S3 and S4. Fig. 9 displays the KEGG pathway enrichment bubble chart, KEGG pathway classification plot, and gene expression clustering heatmap of differentially expressed genes, respectively. Functional enrichment analysis of differentially expressed genes revealed that the anti-inflammatory genes thrombomodulin (THBD) and Ets proto-oncogene 2 (Ets2) were both significantly upregulated in samples from the ZIF-8@CuO/C-VEGFMP-NIR group (Fig. S11). THBD neutralizes high-mobility group box 1 (HMGB1) *via* the lectin-like domain of thrombomodulin (TMD1) to balance M1/M2 macrophages and suppress pro-inflammatory cytokines; it activates signal transducer and activator of transcription 6 (STAT6)/protein kinase B (AKT) signaling pathway to protect endothelium, alleviates scarring, and facilitates angiogenesis and tissue regeneration.^51,52^ Upregulated Ets2 inhibits the phosphorylation of MAPK and NF-κB and reduces the production of pro-inflammatory cytokines. It can also bind to the IL-6 promoter to block its transcription. Animal experiments have confirmed that Ets2 knockout aggravates sepsis and inflammatory responses in mice, verifying its anti-inflammatory function.^53^ Collectively, transcriptomic profiling indicates that upregulated THBD and Ets2 are potentially associated with the therapeutic outcomes of ZIF-8@CuO/C-VEGFMP hydrogel combined with NIR irradiation. Further functional experiments such as gene knockdown or overexpression are required to elucidate their exact causal roles.

**Fig. 9:**
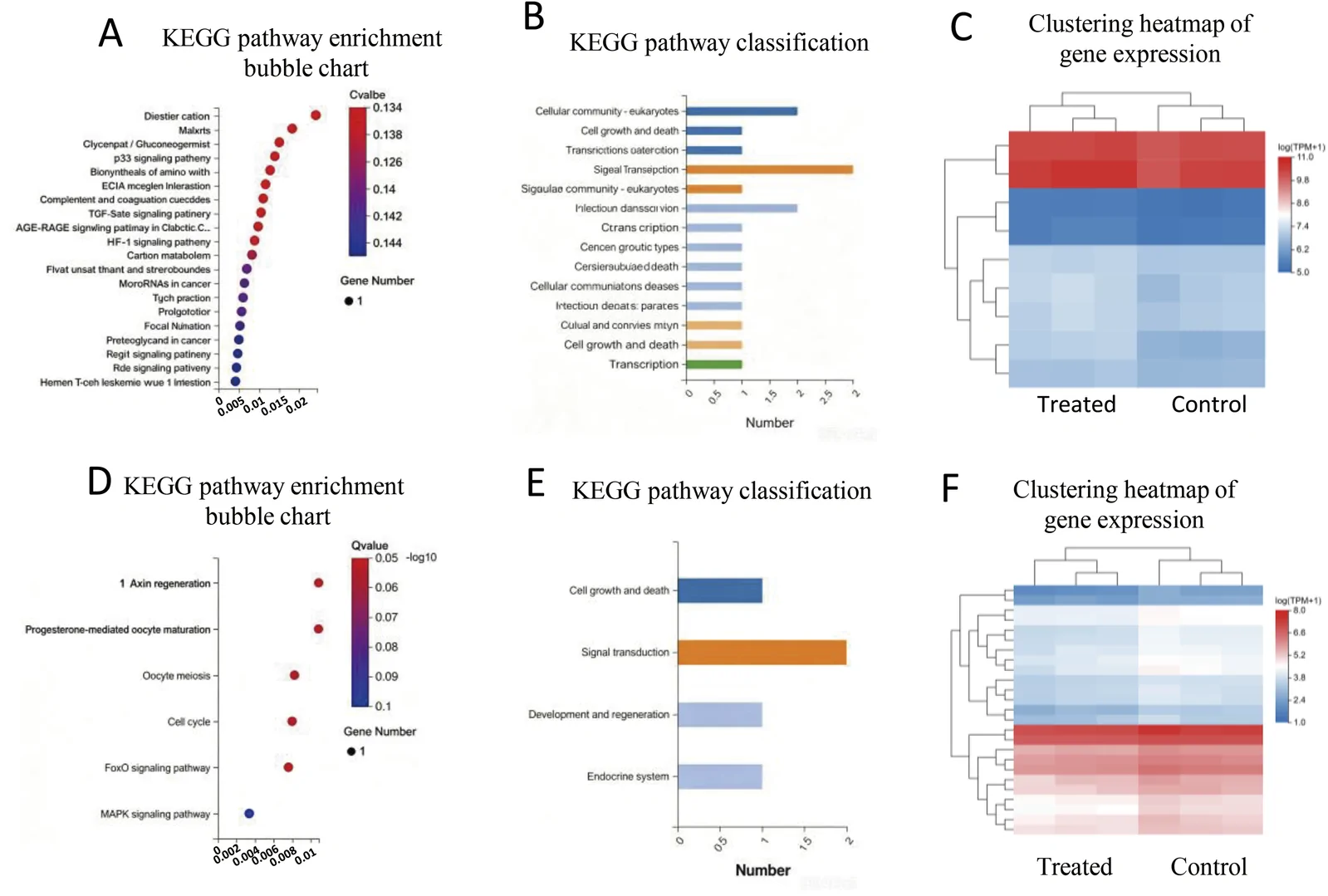
KEGG pathway enrichment analysis and expression clustering heatmaps of upregulated (A–C) and downregulated (D–F) differentially expressed genes.

In this study, a multifunctional ZIF-8@CuO/C-VEGFMP hydrogel was successfully prepared. This hydrogel possessed mild photothermal antibacterial properties, sustained metal-ion release capability, and targeted pro-angiogenic bioactivity. It is suitable for the treatment of full-thickness skin wounds infected with bacteria. The ZIF-8@CuO nanocomposite overcomes the limitations of a single component: ZIF-8 lacks photothermal conversion ability and usually cannot effectively eliminate severe chronic infections. The controllable synthesis of CuO on the surface of ZIF-8 achieves a dual-ion and photothermal synergistic effect, while retaining ZIF-8 bioactivity and effectively inhibiting pathogenic bacteria. Additionally, C-VEGFMP addresses the problem of the rapid release and short half-life of traditional peptides. By introducing specific collagen-binding domains, C-VEGFMP can stably and persistently anchor to the collagen-rich GelMA hydrogel, thereby exerting sustained *in situ* pro-angiogenic effects.

Owing to the synergistic effect of photothermal therapy and zinc- and copper-ion-mediated sterilization, this composite hydrogel can efficiently kill bacteria and destroy bacterial biofilms under precisely controlled NIR irradiation in the early stage of wound infection, while effectively avoiding thermal damage to fragile regenerative skin tissue. Unlike single-function antibacterial dressings that rely solely on high-temperature ablation or physical barrier protection, the hydrogel designed in this study achieves staged and precise wound intervention: intermittent NIR-assisted synergistic sterilization rapidly reduces the bacterial load to control acute infection, followed by the sustained release of zinc and copper ions to achieve long-term antibacterial protection and optimize the wound immune microenvironment. Meanwhile, Zn^2+^ can efficiently reverse M1-type pro-inflammatory macrophage polarization toward the M2-type repair phenotype, thereby reducing excessive inflammatory responses and creating a favorable microenvironment for tissue reconstruction. The anchored C-VEGFMP peptide can sustainably promote endothelial cell proliferation and angiogenesis, further accelerating connective tissue formation, collagen deposition, and epithelial regeneration during the healing process.

The results of *in vivo* histological staining and wound healing experiments consistently confirmed that this synergistic photothermal-multifunctional hydrogel treatment strategy significantly promoted the healing of infected skin wounds and exhibited reliable biosafety. Transcriptomic results indicated that ZIF-8@CuO, C-VEGFMP, and NIR treatment primarily exerted anti-inflammatory, pro-angiogenic, and tissue repair effects by upregulating THBD and Ets2. In summary, this multifunctional composite hydrogel provides a reliable and feasible synergistic treatment strategy for the clinical repair of infectious skin wounds and represents a promising candidate material for advanced dressings.

## 4 Conclusion

In this study, ZIF-8@CuO nanoparticles and the collagen-binding peptide C-VEGFMP were prepared, and a multifunctional hydrogel dressing was fabricated by using these components. The experimental results showed that this multifunctional hydrogel dressing exhibited a porous structure, favorable mechanical properties, biocompatibility, hemostatic ability, antioxidant activity, antibacterial activity, and photothermal conversion capability. The ZIF-8@CuO/C-VEGFMP hydrogel exhibited efficient antibacterial performance under NIR light irradiation. Moreover, C-VEGFMP exhibited stable binding to the hydrogel and sustained its biological activity over time. Cell experiment showed that ZIF-8@CuO/C-VEGFMP hydrogel could enhance cell viability and proliferation. *In vivo* experiments revealed that this hydrogel material combined with photothermal therapy could accelerate the healing of infected skin wounds, alleviate inflammation, promote collagen deposition, and epithelial cell regeneration. Transcriptomic analysis indicated that the ZIF-8@CuO/C-VEGFMP-NIR hydrogel exerts anti-inflammatory, pro-angiogenic and tissue regenerative effects mainly *via* upregulating the THBD and Ets2 genes. Therefore, this multifunctional hydrogel offers a new strategy for combined therapy for tissue regeneration and holds promise for clinical translation in the treatment of infected skin wounds.

## Conflicts of interest

The authors declare that there are no conflicts of interest.

## Supplementary Material

RA-OLF-D6RA06950A-s001

## Data Availability

All analysed and processed data supporting the findings of this study, including material characterization results, *in vitro* cell experimental data, *in vivo* wound-ealing histological images and related quantitative statistics, are fully included within this article and its supplementary Information (SI). Supplementary information is available. See DOI: https://doi.org/10.1039/d6ra06950a.
