# Supplementary material for: A multifunctional ZIF-8@CuO/collagen-binding peptide hydrogel for photothermal-enhanced antibacterial and infected skin wound management
Source: RSC Adv. 2026 Oct 8;16(46):60983–99. doi: 10.1039/d6ra06950a (PMC13647587; doi:10.1039/d6ra06950a)
Supplement: Supplementary file 1 [file RA-016-D6RA06950A-s001.pdf]

Supplementary Information (SI) for RSC Advances.
This journal is © The Royal Society of Chemistry 2026

Supplementary Materials

A multifunctional ZIF-8@CuO/collagen-binding peptide hydrogel for photothermal-enhanced antibacterial and infected skin wound management

Shengyang Li, Renfeng Zhang, Haorui Du, Hanwen Yang, Su Pan*

Department of Orthopedics, the Second Hospital of Jilin University, Changchun, Jilin 130022, China

* Corresponding authors. E-mail addresses: pansu@jlu.edu.cn.

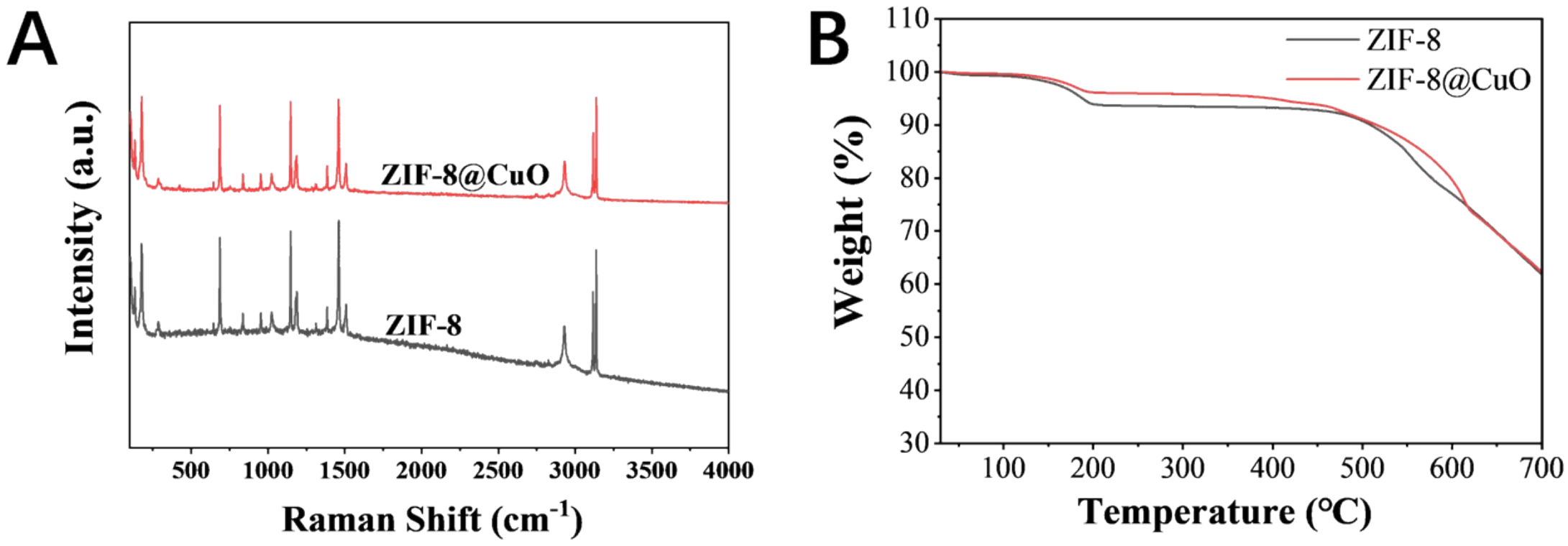

Fig.S1 (A) Raman spectra of ZIF-8 and ZIF-8@CuO. (B) TGA curves of ZIF-8 and ZIF-8@CuO.

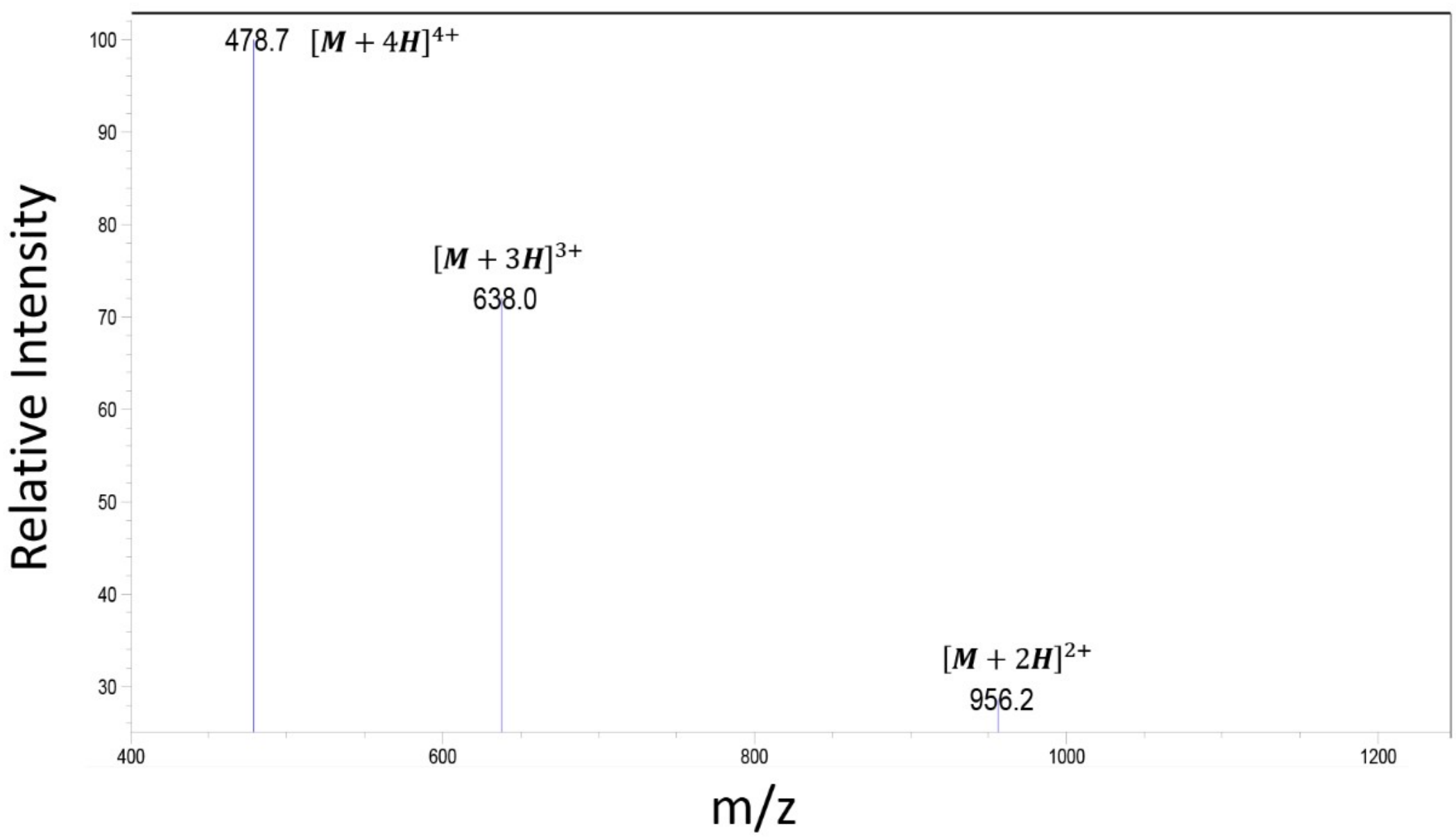

Fig.S2 ESI-MS spectrum of synthesized VEGFMP peptide

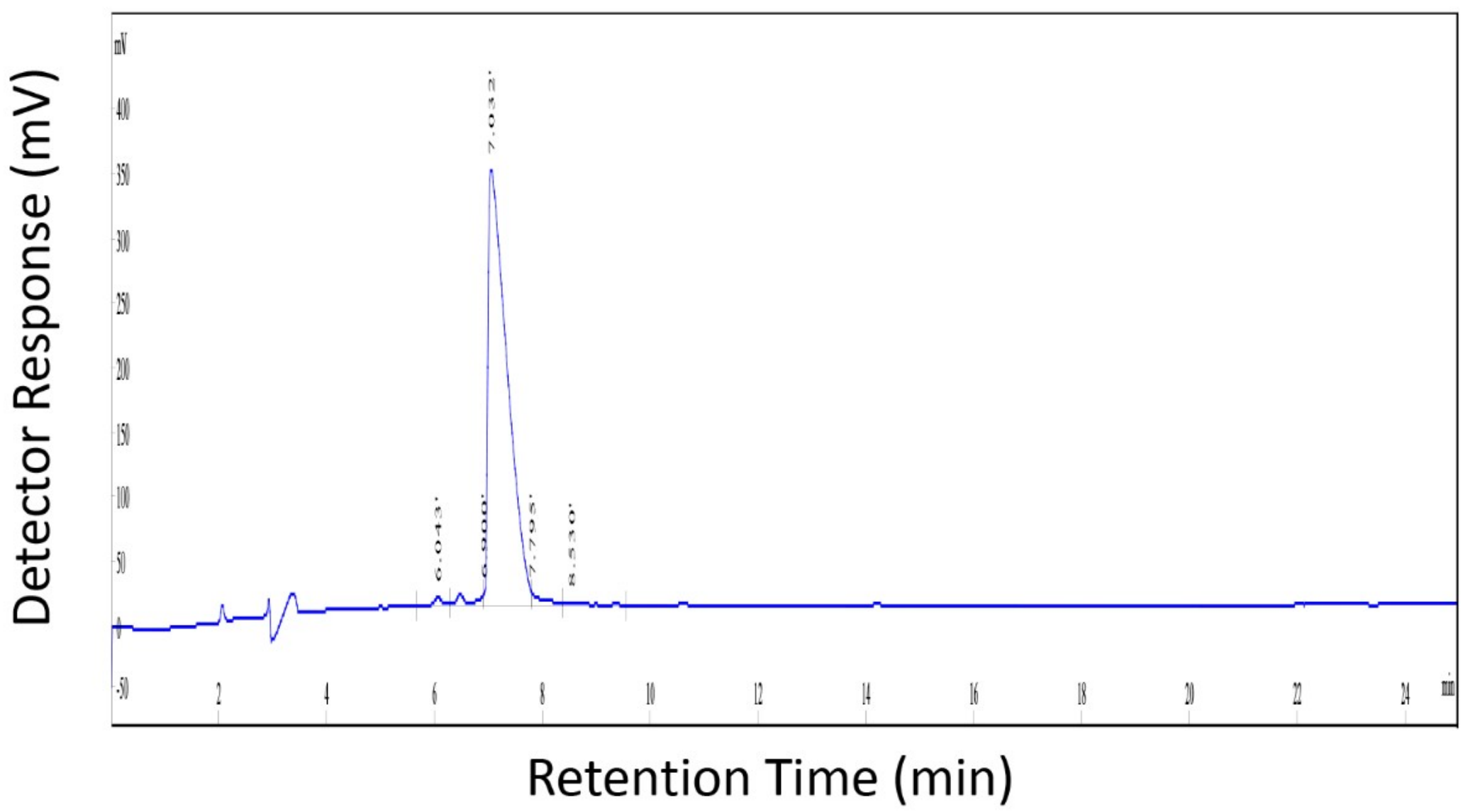

Fig.S3 Purity analysis of VEGFMP peptide by HPLC

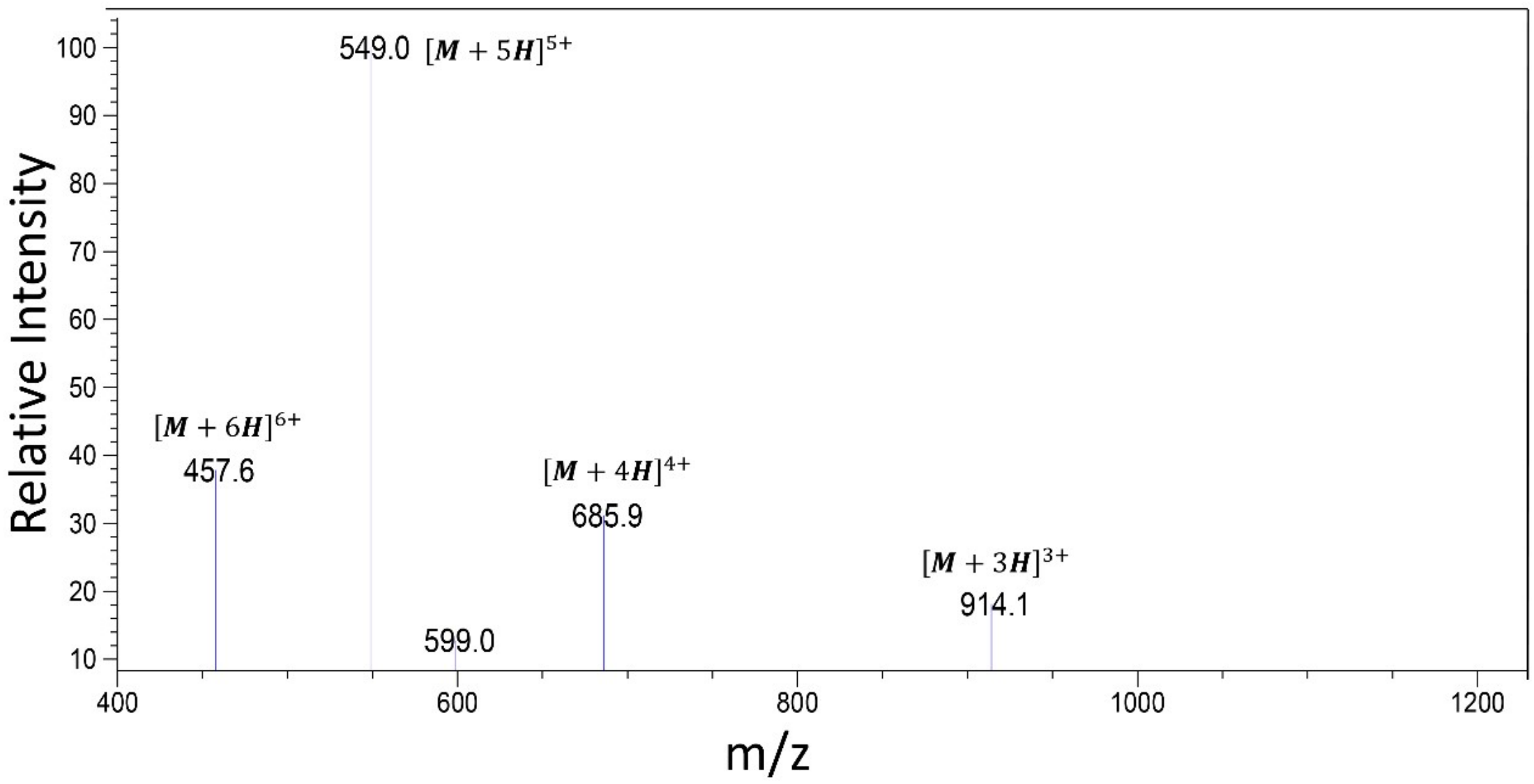

Fig.S4 ESI-MS spectrum of synthesized CVEGFMP peptide.

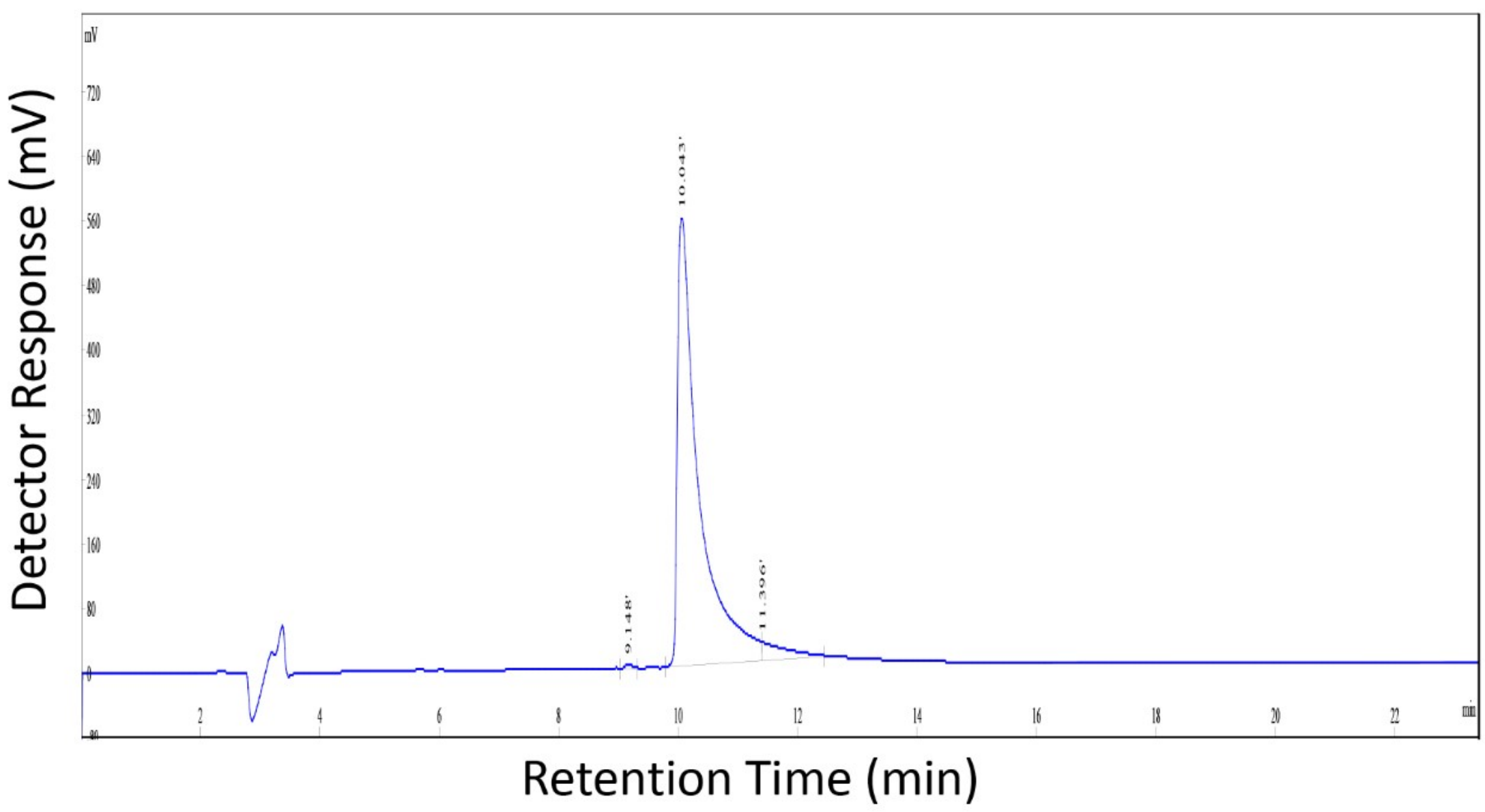

Fig.S5 Purity analysis of VEGFMP peptide by HPLC

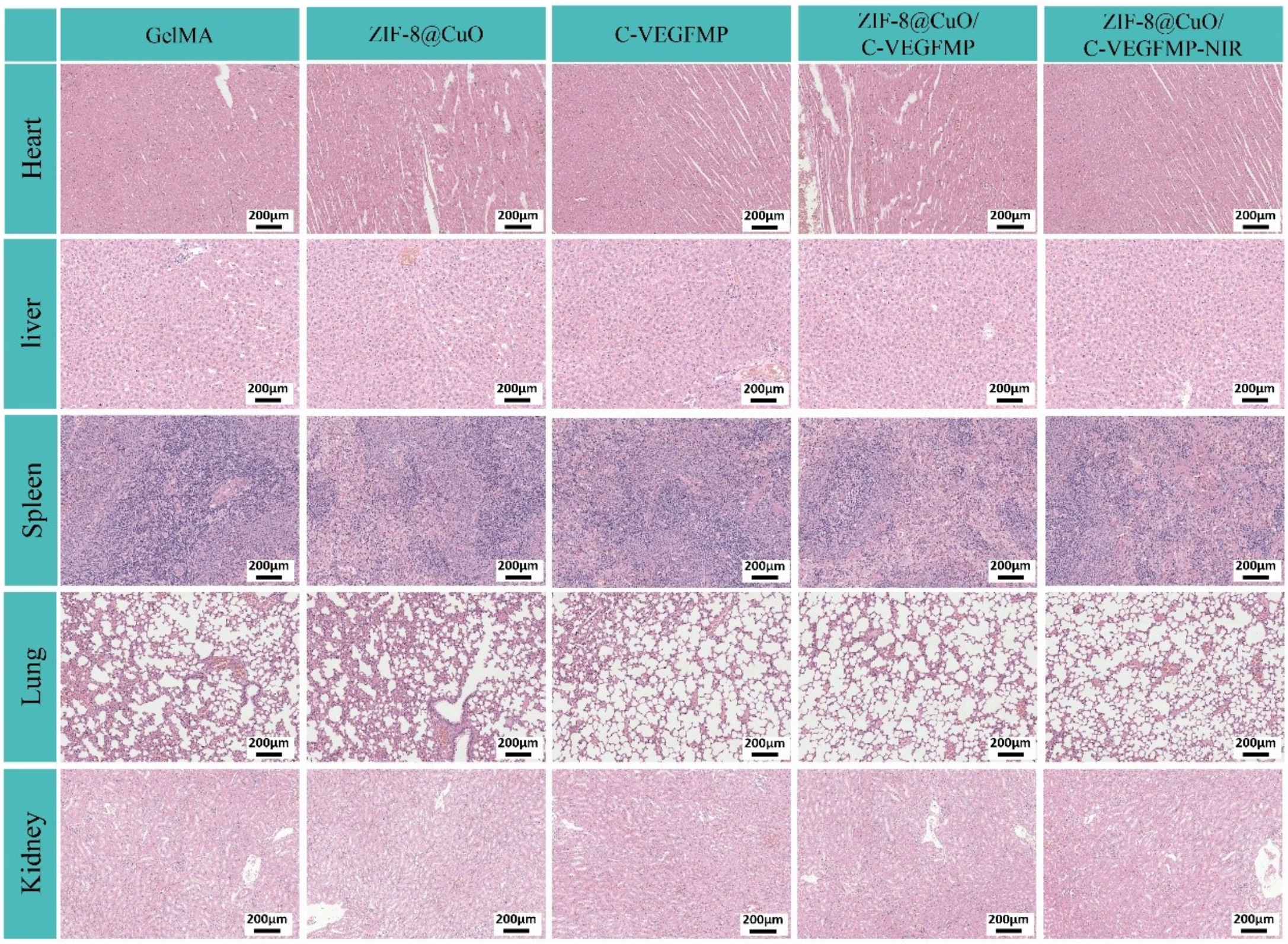

Fig.S6. Histological H&E images of rat major organs in various hydrogel groups.

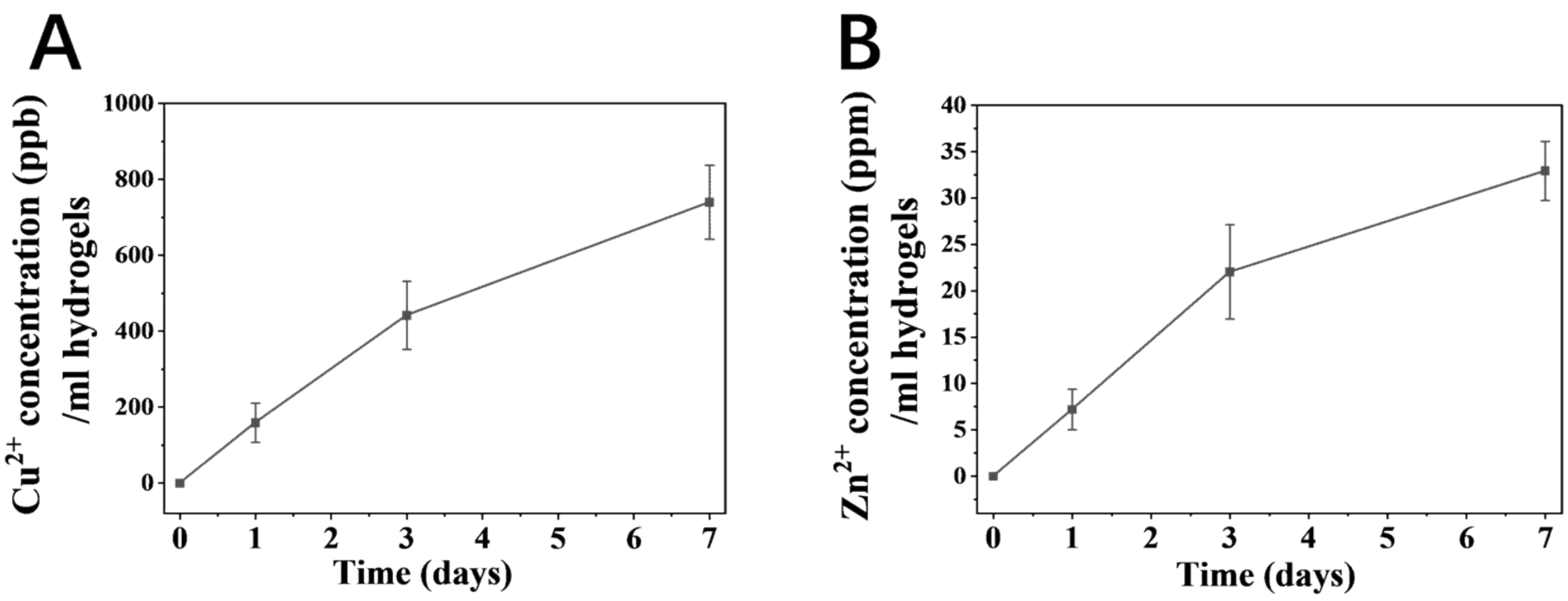

Fig.S7 Release curves of Cu^{2+} (A) and Zn^{2+} (B) from ZIF-8@CuO hydrogels during 7 days of incubation.

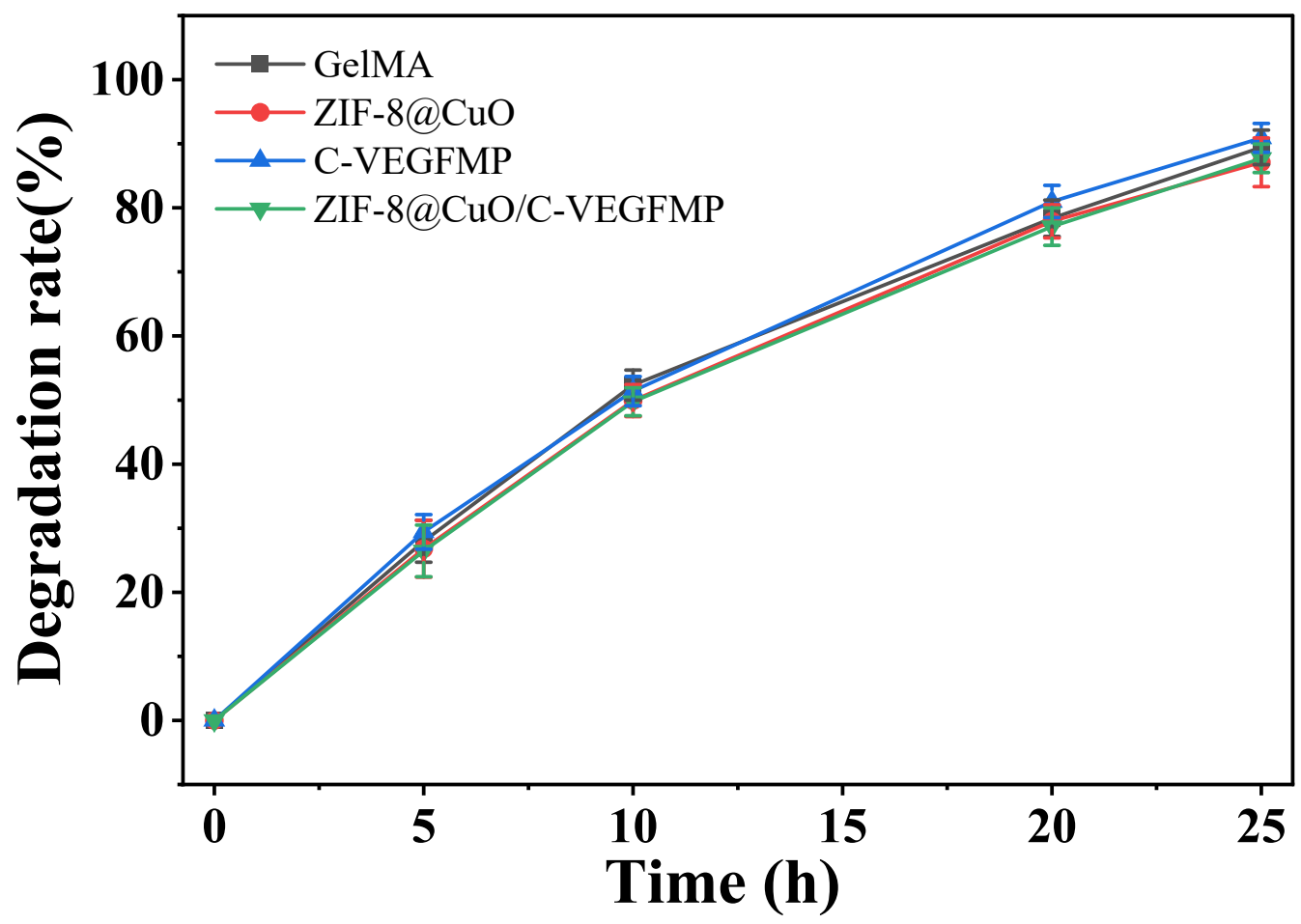

Fig.S8 Degradation rate the hydrogels in PBS containing 5 U/mL type II collagenase.

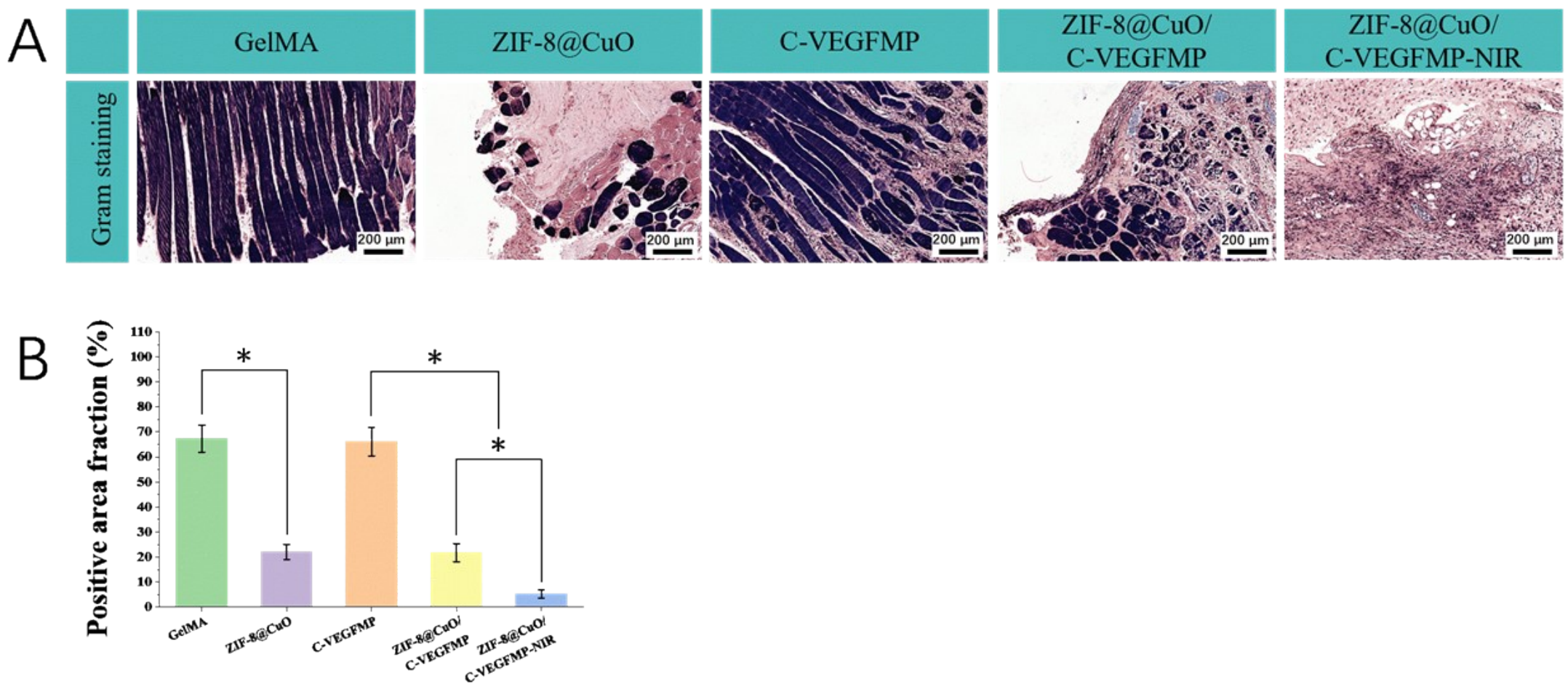

Fig. S9 Gram staining of infected wound tissues harvested on day 3 after hydrogel implantation. (A) Representative Gram staining micrographs of tissue sections for different treatment groups. Scale bar = 200 μm. (B) Quantitative statistics of the Gram-positive bacterial positive area fraction, n=3 *p<0.05.

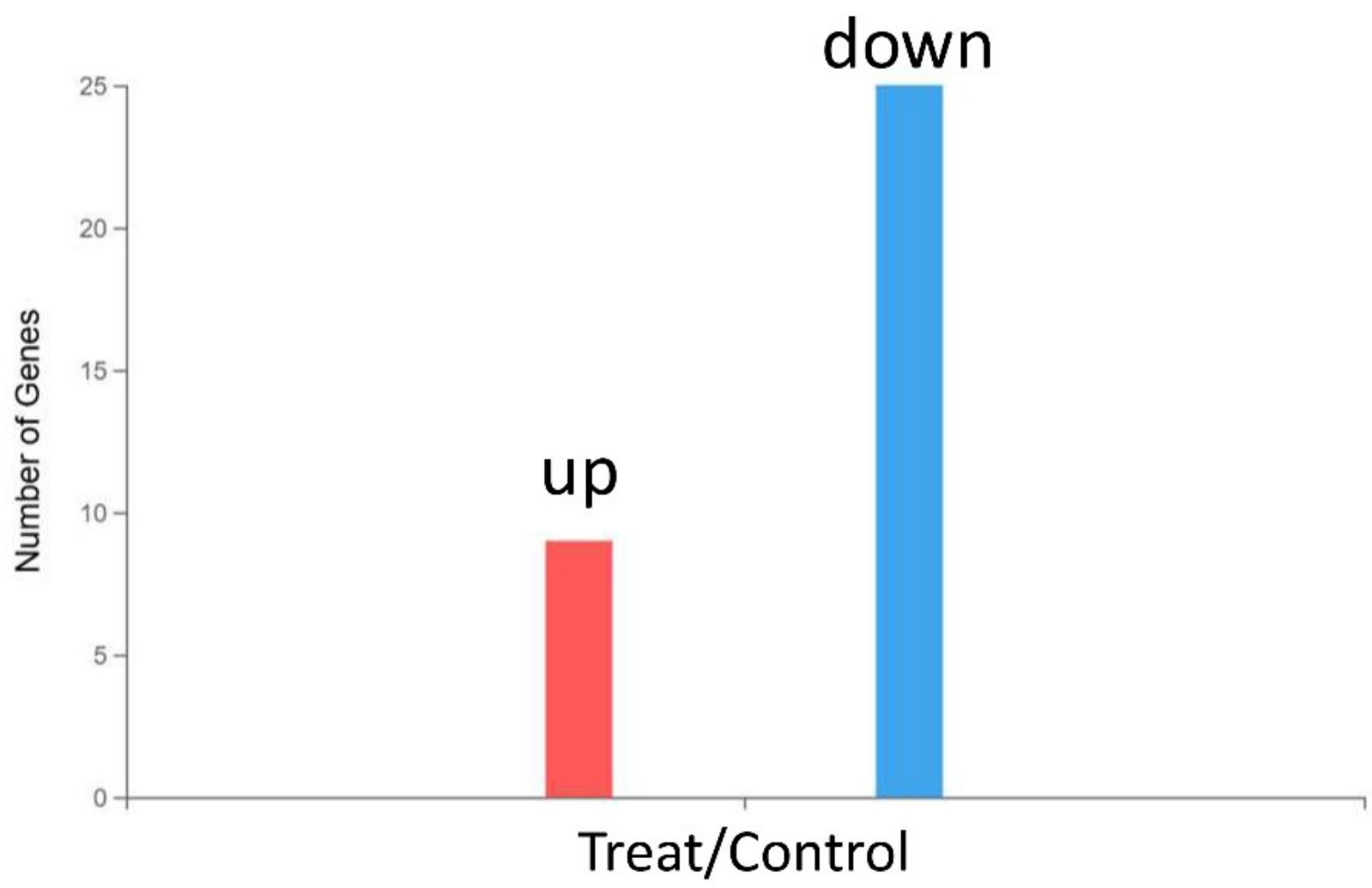

Fig.S10 Statistics of the number of differentially expressed Genes (ALL)

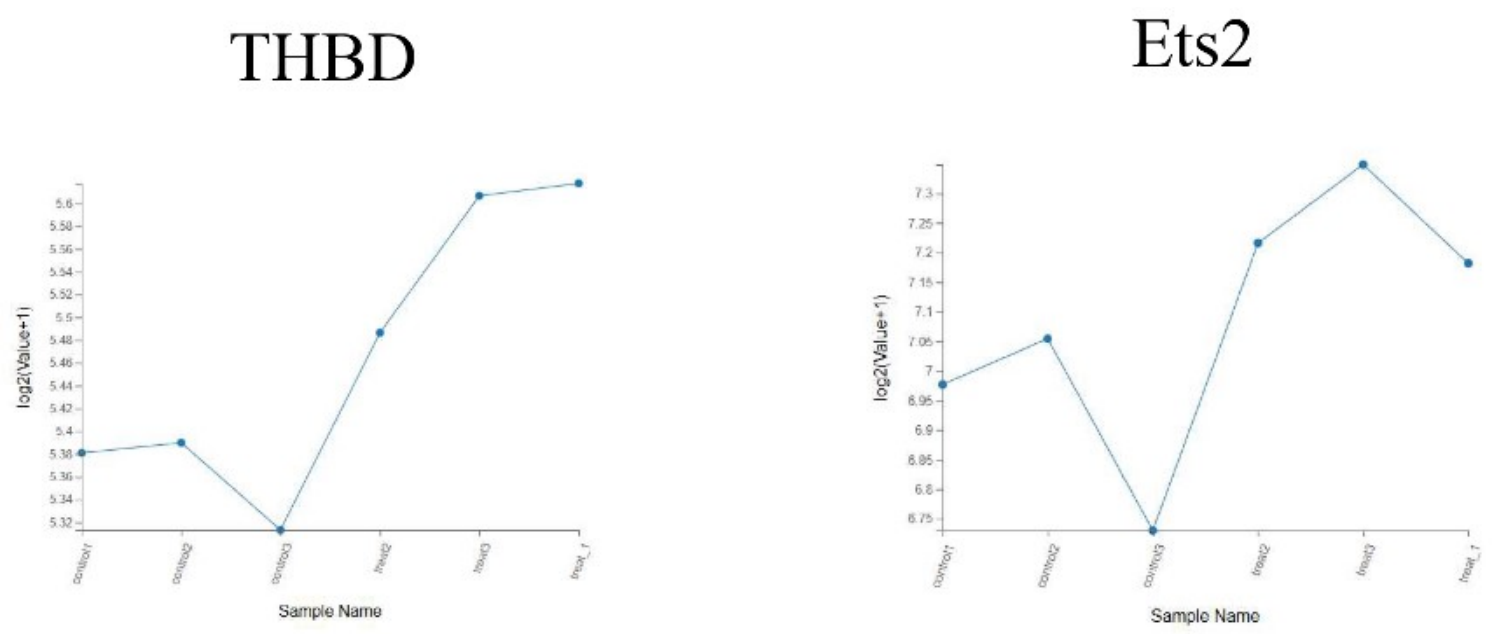

Fig.S11 Expression profiles of THBD and Ets2 genes across control and treatment samples.

Table S1. Primer sequences for qPCR analysis of mouse genes

Gene name		Primer sequence（5‘-3’）
TNFα	F:	GACGTGGAACTGGCAGAAGAG
	R:	TTGGTGGTTTGTGAGTGTGAG
IL-6	F:	GAGGATACCACTCCCAACAGACC
	R:	AAGTGCATCATCGTTGTTCATACA
IL-10	F:	GGTTGCCAAGCCTTATCGGA
	R:	ACCTGCTCCACTGCCTTGCTCTT
Arg-1	F:	CTCCAAGCCAAAGTCCTTAGAG
	R:	AGGAGCTGTCATTAGGGACATC
CD31	F:	TTGAGCCTCACCAAGAGAACGGAA
	R:	AATCCAGCTTGCTTTCTTGGGTGTC
COL-I	F:	GCTCCTCTTAGGGGCCACT
	R:	CCACGTCTCACCATTGGGG
GAPDH	F:	CATGGCCTTCCGTGTTCCTA
	R:	GCGGCACGTCAGATCCA

Table S2. Serum biochemical parameters of rats at day 21 after subcutaneous implantation of different hydrogels.

Item	GelMA	ZIF-8@CuO	C-VEGFMP	ZIF-8@CuO/C-VEGFMP	Unit	Reference Range
TP	65.6	66.1	64.9	65.8	g/L	48.0-69.0
ALB	39.3	39.5	38.1	38.4	g/L	33.0-44.0
GLOB	23.3	26.6	26.5	24.9	g/L	18.0-32.0
A/G	1.3	1.5	1.6	1.5	-	-
ALT	46.7	48.4	49.6	47.1	U/L	0.0-90.0
AST	89.5	82.2	91.4	85.7	U/L	0.0-180.0
AST/ALT	1.92	1.7	1.84	1.82	-	-
ALP	242.5	237.8	239.6	243.1	U/L	0.0-400.0
GGT	<2.0	<2.0	<2.0	<2.0	U/L	0.0-5.0
TBIL	<1.70	<1.70	<1.70	<1.70	μmol/L	0.00-10.00
TBA	11.7	11.4	11.9	40	μmol/L	-
AMY	1672	1634.6	1886.7	1909.3	U/L	300.0-3041.0
BUN	0.8	0.74	0.67	0.79	mmol/L	0.61-1.61
CREA	37.2	32.5	33.8	33.4	μmol/L	8.00-50.00
BUN/CREA	78.5	76.1	78.1	75.5	-	-
CK	545.2	546.6	487.4	542.6	U/L	0.0-920.0
LDH	690.2	688.7	657.4	673.2	U/L	0.0-920.0
GLU	7.25	7.45	7.59	7.26	mmol/L	4.40-10.50
TC	1.97	2.05	2.06	2.06	mmol/L	0.70-2.34
TG	0.85	0.78	1.01	0.89	mmol/L	0.20-1.87
Ca	2.22	2.71	2.67	2.82	mmol/L	2.20-2.95
PHOS	2.97	2.17	2.77	2.42	mmol/L	1.00-3.93
CaxP	6.59	5.88	7.4	6.82	mmol/L^2	-
Mg	0.81	0.86	0.85	0.84	mmol/L	0.70-1.33
Na^+	146.7	145.9	145.2	143.6	mmol/L	140.0-147.0
K^+	6.2	5.4	6.1	5.8	mmol/L	3.3-6.2
Na/K	23.7	27	23.8	24.8	-	-
Cl^-	102.4	103.9	101.3	105.2	mmol/L	98.0-110.0

Table. S3 Genes with upregulated expression.

Gene ID	Gene Symbol	Type	log2 (treat / control)	Qvalue (treat / control)
16601	'Klf9'	mRNA	0.238192095	0.016020685
17937	'Nab2'	mRNA	0.25040989	4.38E-04
18655	'Pgk1'	mRNA	0.200510493	0.03709619
21824	'Thbd'	mRNA	0.249915614	0.033274353
21825	'Thbs1'	mRNA	0.347754932	0.001020194
23872	'Ets2'	mRNA	0.362109888	0.001769373
50708	'H1f2'	mRNA	0.62410299	1.32E-08
56332	'Amotl2'	mRNA	0.245531252	0.044909393
70717	'Medag'	mRNA	0.225074114	0.009962755

Table. S4 Genes with downregulated expression

Gene ID	Gene Symbol	Type	log2 (treat / control)	Qvalue (treat / control)
108000	'Cenpf'	mRNA	-0.325325128	0.013378645
108912	'Cdca2'	mRNA	-0.34224434	0.046906589
12316	'Aspm'	mRNA	-0.352567116	0.004922834
12449	'Ccnf'	mRNA	-0.351896795	0.013378645
16551	'Kif11'	mRNA	-0.375395476	1.03E-04
17345	'Mki67'	mRNA	-0.394928339	1.94E-04
18817	'Plk1'	mRNA	-0.277422673	0.043277372
208084	'Pif1'	mRNA	-0.531729754	0.005502775
214791	'Sertad4'	mRNA	-0.351195838	0.033274353
21841	'Tia1'	mRNA	-0.247632828	0.013378645
229841	'Cenpe'	mRNA	-0.314199387	4.33E-04
240641	'Kif20b'	mRNA	-0.451510275	5.79E-05
26404	'Map3k12'	mRNA	-0.369238224	0.005727861
27981	'Rsrp1'	mRNA	-0.274506727	0.037907294
320558	'Sycp2'	mRNA	-0.619387029	0.005361575
381280	'Hjurp'	mRNA	-0.310007999	1.03E-04
68298	'Ncapd2'	mRNA	-0.30785395	8.73E-04
68549	'Sgo2a'	mRNA	-0.439737752	1.94E-04
71514	'Sfpq'	mRNA	-0.212702418	0.005924544
71878	'Fam83d'	mRNA	-0.528356194	0.005924544
72739	'Zkscan3'	mRNA	-0.366859429	0.001769373
73804	'Kif2c'	mRNA	-0.457899229	8.40E-05
75202	'Spaca6'	mRNA	-0.454494638	0.046906589
75317	'Parpbp'	mRNA	-0.386390885	0.023821891
76464	'Knl1'	mRNA	-0.365351984	0.015259466
